# The crystal structures and Hirshfeld surface analysis of 6-(naphthalen-1-yl)-6a-nitro-6,6a,6b,7,9,11a-hexa­hydro­spiro­[chromeno[3′,4′:3,4]pyrrolo­[1,2-*c*]thia­zole-11,11′-indeno­[1,2-*b*]quinoxaline] and 6′-(naphthalen-1-yl)-6a′-nitro-6′,6a′,6b′,7′,8′,9′,10′,12a′-octa­hydro-2*H*-spiro­[ace­naphthyl­ene-1,12′-chromeno[3,4-*a*]indolizin]-2-one

**DOI:** 10.1107/S205698901901291X

**Published:** 2019-09-27

**Authors:** G. Foize Ahmad, A. Syed Mohammed Mujaheer, M. NizamMohideen, M. Gulam Mohamed, V. Viswanathan

**Affiliations:** a PG & Research Department of Physics, The New College (Autonomous), University of Madras, Chennai 600 014, Tamil Nadu, India; b Department of Biophysics, All India Institute of Medical Science, New Delhi 110029, India

**Keywords:** crystal structure, nitro­gen-containing heterocycles, chromen, spiro compounds, cyclo­addition, piperidine, pyran, pyrrolidine, hydrogen bonding, C—H⋯π inter­actions, Hirshfeld surface analysis

## Abstract

The title compounds, 6-(naphthalen-1-yl)-6a-nitro-6,6a,6 b,7,9,11*a*-hexa­hydro­spiro­[chromeno[3′,4′:3,4]pyrrolo­[1,2-*c*]thia­zole-11,11′-indeno­[1,2-*b*]quinoxaline], C_37_H_26_N_4_O_3_S, (I), and 6′-(naphthalen-1-yl)-6a′-nitro-6′,6a′,6b′,7′,8′,9′,10′,12*a*′-octa­hydro-2*H*-spiro­[ace­naphthyl­ene-1,12′-chromeno[3,4-*a*]indolizin]-2-one, C_36_H_28_N_2_O_4_, (II), are new spiro derivatives, in which both the pyrrolidine rings adopt twisted conformations. In (I), the five-membered thia­zole ring adopts an envelope conformation, while the eight-membered pyrrolidine-thia­zole ring adopts a boat conformation. An intra­molecular C—H⋯N hydrogen bond occurs, involving a C atom of the pyran ring and an N atom of the pyrazine ring. In (II), the six-membered piperidine ring adopts a chair conformation. An intra­molecular C—H⋯O hydrogen bond occurs, involving a C atom of the pyrrolidine ring and the keto O atom. For both compounds, the crystal structure is stabilized by inter­molecular C—H⋯O hydrogen bonds. In (I), the C—H⋯O hydrogen bonds link adjacent mol­ecules, forming *R*_2_^2^(16) loops propagating along the *b*-axis direction, while in (II) they form zigzag chains along the *b*-axis direction. In both compounds, C—H⋯π inter­actions help to consolidate the structure, but no significant π–π inter­actions with centroid–centroid distances of less than 4 Å are observed.

## Abstract 2

The crystal structures of the title spiro derivatives are described and the analysis of the inter­molecular contacts in the crystals using Hirshfeld surface analysis and two-dimensional fingerprint plots is reported.

## Chemical context   

Nitro­gen-containing heterocycles and their derivatives are present in many large mol­ecules suitable for photo-chemical, electrochemical and catalytic applications; moreover, some derivatives also possess non-linear optical (NLO) properties (Babu *et al.*, 2014*a* ▸,*b* ▸). Spiro compounds are potential precursors for biologically important compounds such as amino sugars (NizamMohideen *et al.*, 2009*a* ▸; Ali *et al.*, 1988 ▸), alkaloids (NizamMohideen *et al.*, 2009*c* ▸; Goti *et al.*, 1997 ▸), and exhibit anti­bacterial and anti­fungal activities (Ravi Kumar *et al.*, 2003 ▸). The 1,3-dipolar cyclo­addition of nitro­nes with olefinic dipolarophiles proceeds through a concerted mechanism yielding highly substituted heterocyclic compounds (Gothelf & Jørgensen, 1998 ▸). The cornerstone for cyclo­addition reactions, nitro­nes, are excellent for spin trapping (NizamMohideen *et al.*, 2009*b* ▸; Bernotas *et al.*, 1996 ▸) and are highly versatile synthetic inter­mediates (Breuer, 1982 ▸). The stereochemistry, such as regioselectivity and enanti­oselectivity, of heterocyclic compounds (Huisgen, 1984 ▸) can be studied by 1,3-dipolar cyclo­addition reactions. Against this background and considering the importance of their natural occurrence, biological, pharmacological and medicinal activities, use as synthetic inter­mediates, as well as in view of our ongoing research on the design of novel heterocycles, we have synthesized the title compounds and report herein their crystal structures.
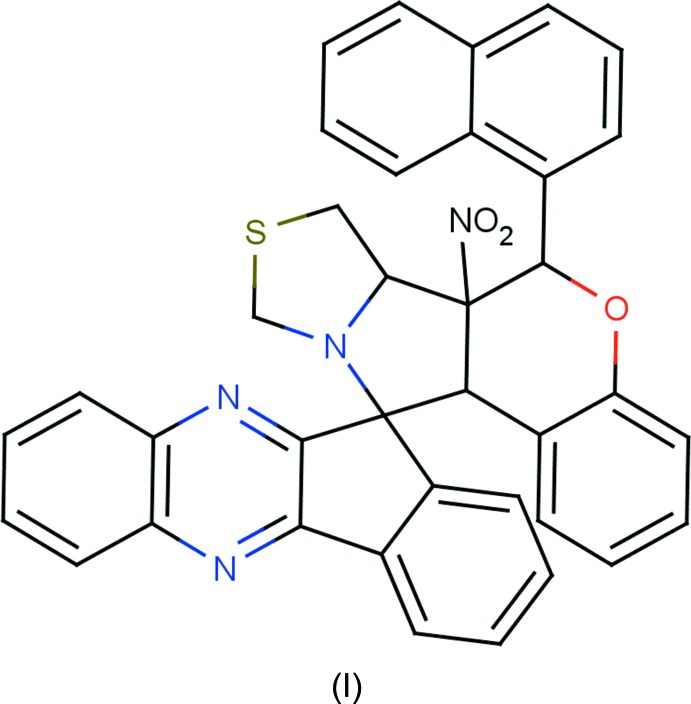
 
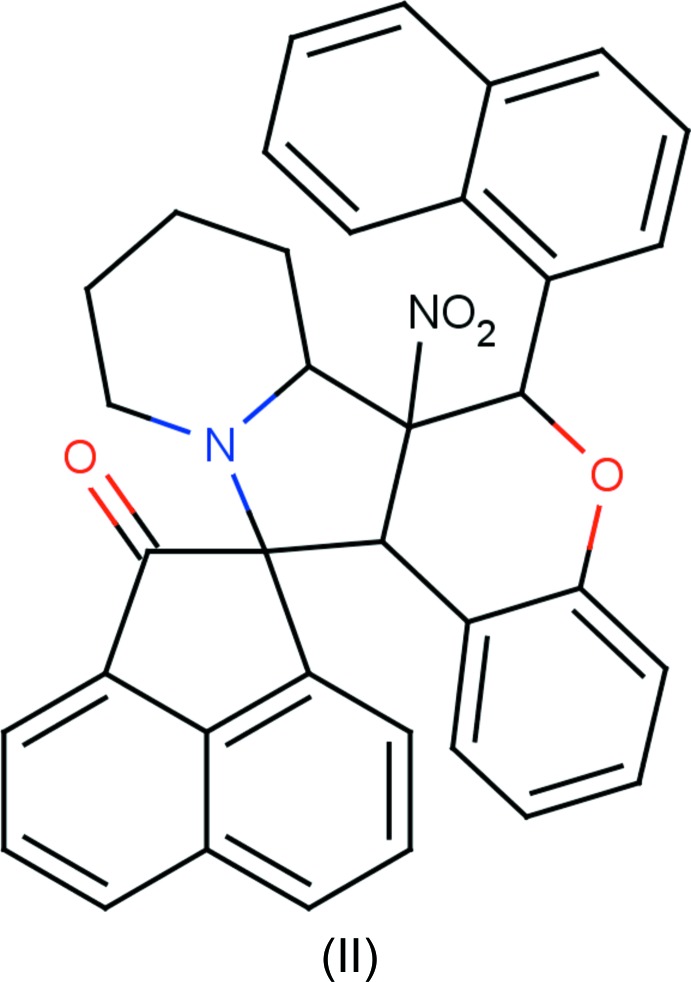


## Structural commentary   

The bond lengths and angles are close to those reported for similar compounds (Devi *et al.*, 2013*a* ▸,*b* ▸; Syed Abuthahir *et al.*, 2019*a* ▸,*b* ▸). In both compounds, the five-membered pyrrolidine ring (N3/C1/C16/C24/C25) adopts a twisted conformation [on C24 and C25 in (I) and on C17 and N1 in (II)], with a pseudo-twofold axis passing through the N3—C1 and N1—C12 bonds, respectively. The puckering parameters are: *q*_2_ = 0.357 (2) Å, φ = 307.0 (3)° for (I) and *q*_2_ = 0.415 (2) Å, φ = 348.5 (3)° for (II). The mean plane of the pyrrolidine ring is almost perpendicular to the mean plane of the cyclo­pentene ring (C1/C2/C7/C8/C15), being inclined by 88.5 (2) in (I) and 84.3 (2)° in (II). It forms dihedral angles of 57.7 (2) in (I) and 63.0 (2)° in (II) with the mean plane of the pyran ring (O1/C16/C17/C22–C24), and subtends dihedral angles of 24.2 (2) in (I) and 45.3 (2)° in (II) with the mean plane of the naphthalene ring system (C28–C37). The mean plane of the pyran ring is inclined to the mean plane of the cyclo­pentene ring by 55.2 (2) in (I) and 36.7 (2)° in (II), while it subtends dihedral angles of 64.3 (2) in (I) and 81.0 (2)° in (II) with the mean plane of the naphthalene unit.

**Figure 1:**
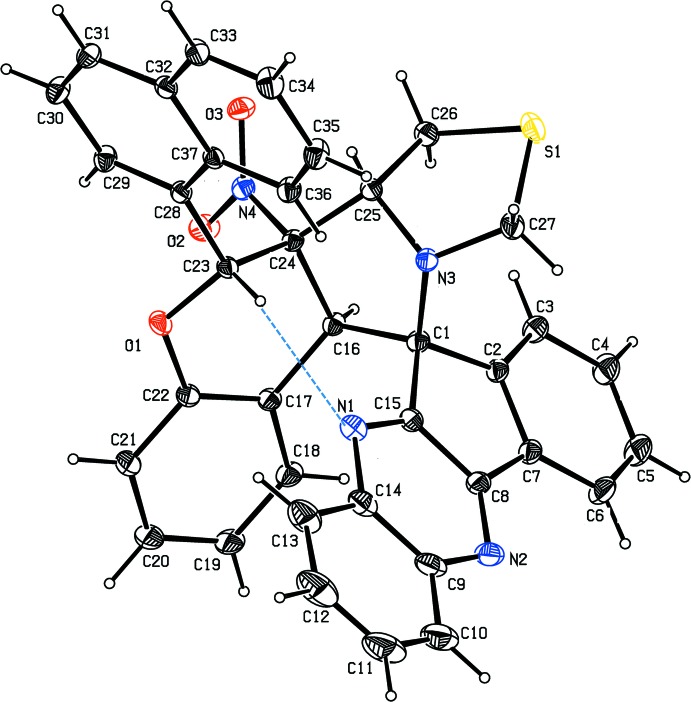
The mol­ecular structure of (I), with atom labelling. Displacement ellipsoids are drawn at the 30% probability level. The intra­molecular C—H⋯N hydrogen bond (Table 1 ▸) is shown as a dashed line.

In (I), the five-membered thia­zole ring (S1/C25–C27/N3) adopts an envelope conformation on C25 with a pseudo-twofold axis passing through the S1—C26 bond. Its puckering parameters are *q*_2_ = 0.391 (2) Å and φ = 251.9 (3)°. The eight-membered pyrrolidine-thia­zole ring (S1/C24–C27/C1/C16/N3) adopts a boat conformation with a total puckering amplitude *Q* = 1.351 (2) Å and φ = 321.43 (8)°. The mean planes of the pyran and thia­zole rings are inclined to each other by 77.5 (2)°. The mean plane of the pyrazine ring (N1/N2/C8/C9/C14/C15) forms a dihedral angle of 57.1 (2)° with the mean plane of the pyran ring, while it is almost perpendicular with respect to the mean plane of the pyrrolidine ring, forming an angle of 89.8 (2)°. The pyrazine ring is inclined by 51.9 (2), 1.9 (2) and 69.5 (2)° with respect to the mean planes of the thia­zole and cyclo­pentene ring and the naphthalene ring system, respectively. An intra­molecular C23–H23⋯N1 hydrogen bond is formed (Fig. 1 ▸).

**Figure 2:**
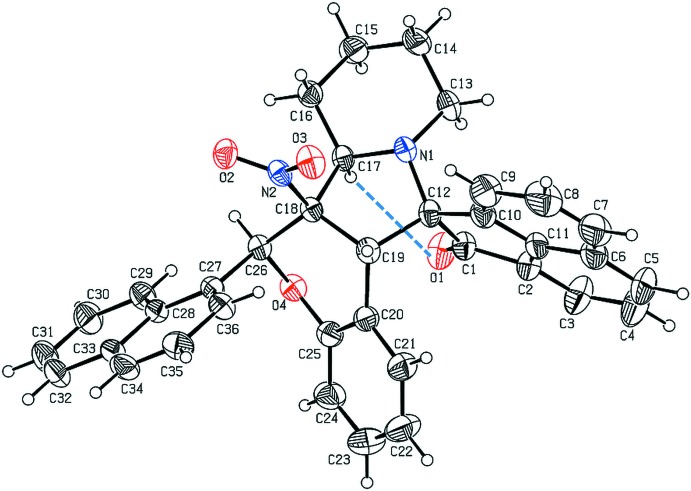
The mol­ecular structure of (II), with atom labelling. Displacement ellipsoids are drawn at the 30% probability level. The intra­molecular C—H⋯O hydrogen bond (Table 2 ▸) is shown as a dashed lines.

In (II), the six-membered piperidine ring (N1/C13–C17) adopts a chair conformation with puckering parameters *q*_2_ = 0.045 (2) Å, θ = 175.7 (2)° and φ = 22 (3)°. The dihedral angle between the ace­naphthyl­ene (C1–C12) and naphthalene (C27–C36) ring systems is 63.8 (6)°. Moreover, this moiety is inclined of 85.3 (1), 36.1 (1) and 89.4 (2) ° with respect to the mean planes of the pyrrolidine (N1/C12/C17–C19), pyran (O4/C18–C20/C25/C26) and piperidine (N1/C13–C17) rings, respectively. The keto atom O1 deviates from the mean plane of the ace­naphthyl­ene unit by 0.148 (1) Å. An intra­molecular C17—H17⋯O1 hydrogen bond is present (Fig. 2 ▸).

## Supra­molecular features   

**Figure 3:**
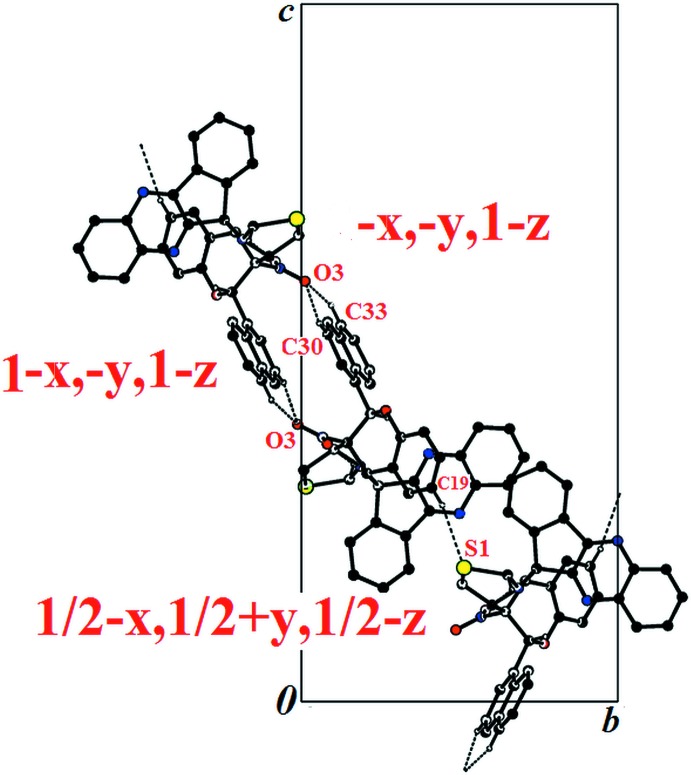
View of the crystal packing of (I) along the *a* axis of the unit cell; only the H atoms involved in the weak inter­actions have been included. In this orientation, the atom O3 in position 1 − *x*, −*y*, 1 − *z* is exactly superimposed on the O3 atom in position −*x*, −*y*, 1 − *z*, which inter­acts with C33—H33. The mol­ecule in position −*x*, −*y*, 1 − *z* is not shown for clarity.

For both compounds, the crystal structure is stabilized by inter­molecular C—H⋯O hydrogen bonds (Tables 1 ▸ and 2 ▸). In (I), the C—H⋯O hydrogen bonds link adjacent mol­ecules, forming 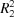
(16) loops propagating along the *b*-axis direction. The loops are linked by C—H⋯S hydrogen bonds, forming layers parallel to the (101) plane; C—H⋯π inter­actions are present within the layers (Table 1 ▸, Fig. 3 ▸).

**Figure 4:**
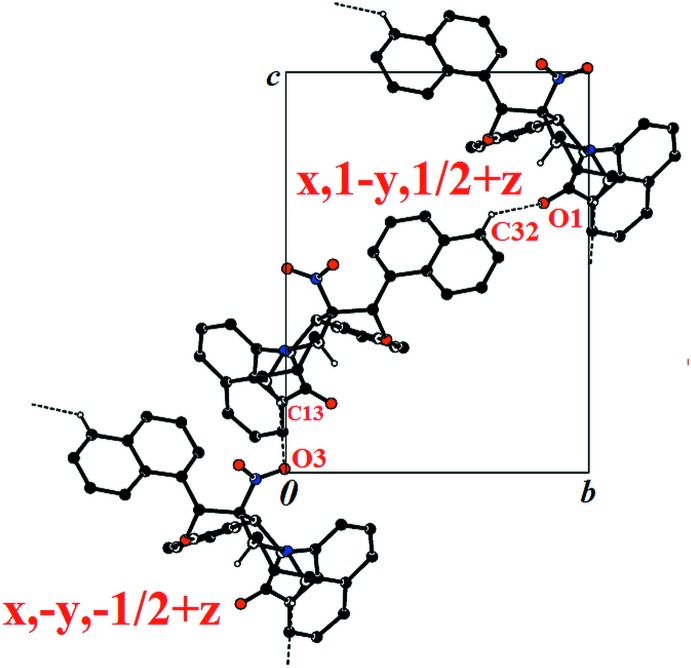
View of the crystal packing of (II) along the *a* axis of the unit cell; only the H atoms involved in hydrogen bonding have been included.

In the crystal of (II), mol­ecules are linked by C—H⋯O inter­actions, forming zigzag chains along the *b*-axis direction (Fig. 4 ▸ and Table 2 ▸). A C—H⋯π inter­action links the chains to form layers parallel to (100), yielding a three-dimensional supra­molecular structure. No significant π–π inter­actions with centroid–centroid distances of less than 4 Å were observed in either compound.

## Hirshfeld surface analysis   

The Hirshfeld surface analysis (Spackman & Jayatilaka, 2009 ▸), and the associated two-dimensional fingerprint plots (McKinnon *et al.*, 2007 ▸), employed to analyse the inter­molecular contacts in the crystals, were performed with *CrystalExplorer17* (Turner *et al.*, 2017 ▸).

**Figure 5:**
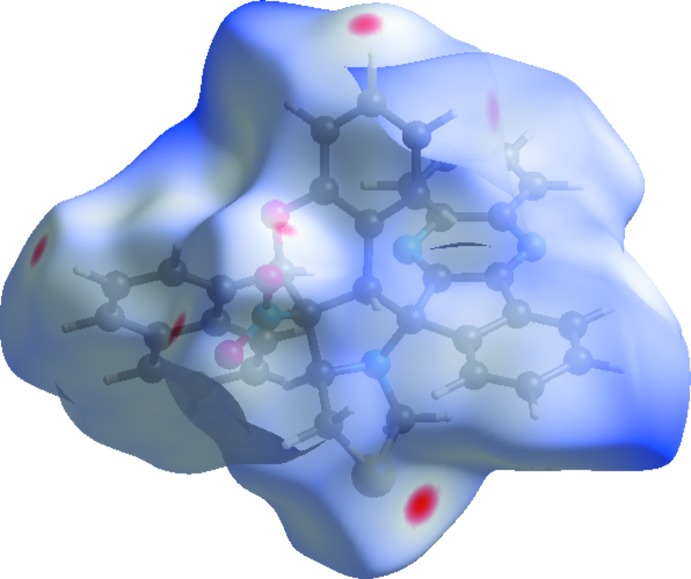
The Hirshfeld surface mapped over *d*_norm_ for (I) mapped over an arbitrary colour scale of −0.177 (red) to 3.260 (blue).

**Figure 7:**
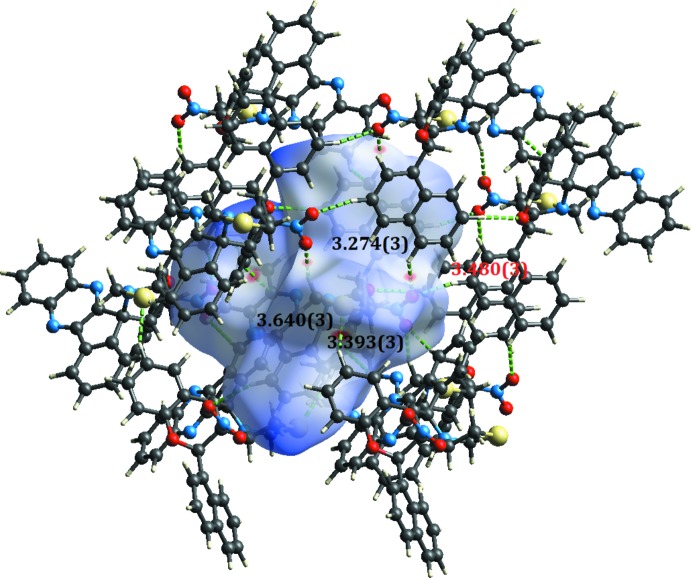
A view of the Hirshfeld surface mapped over *d*_norm_ for (I), showing the various inter­molecular contacts in the crystal.

**Figure 8:**
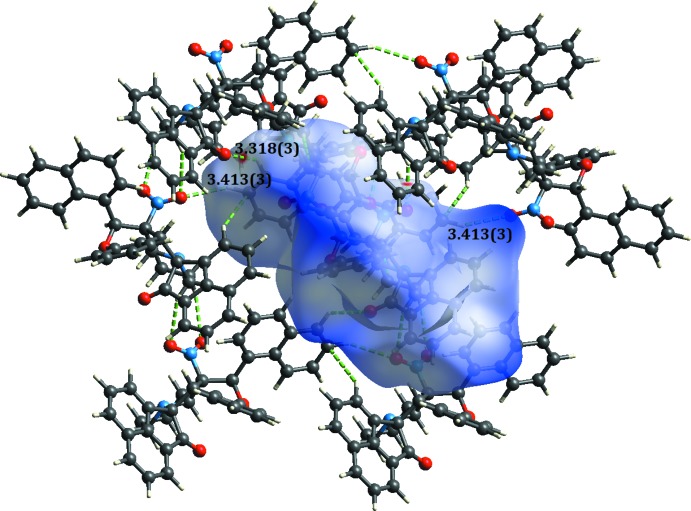
A view of the Hirshfeld surface mapped over *d*_norm_ for (II), showing the various inter­molecular contacts in the crystal.

The Hirshfeld surfaces of (I) and (II) mapped over *d*_norm_ are given in Figs. 5 ▸ and 6 ▸, respectively, while the inter­molecular contacts are illustrated in Fig. 7 ▸ for (I) and in Fig. 8 ▸ for (II). They are colour-mapped with the normalized contact distance, *d*_norm_, varying from red (distances shorter than the sum of the van der Waals radii) through white to blue (distances longer than the sum of the van der Waals radii). The red spots on the surface indicate the inter­molecular contacts involved in hydrogen bonding.

**Figure 9:**
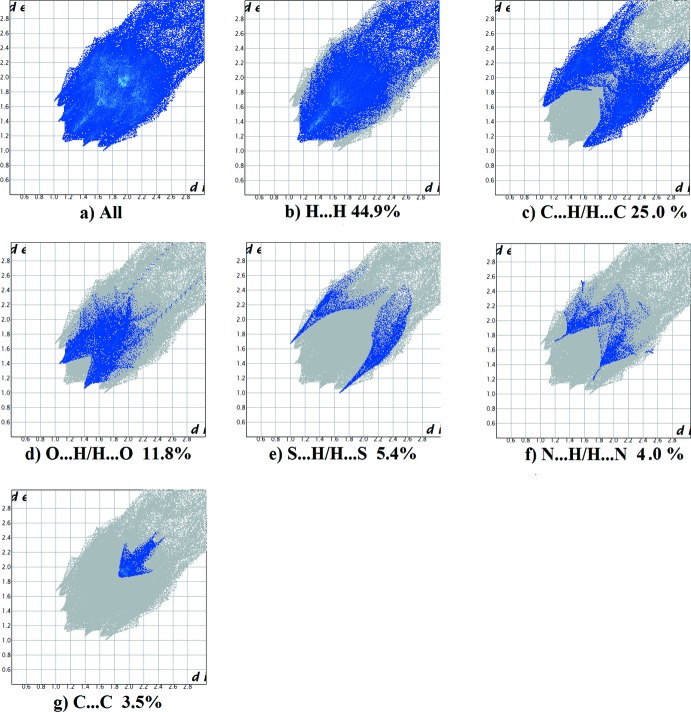
The full two-dimensional fingerprint plot for (I) (*a*), and the fingerprint plots delineated into (*b*) H⋯H, (*c*) C⋯H/H⋯C, (*d*) O⋯H/H⋯O, (*e*) S⋯H/H⋯S, (*f*) N⋯H/H⋯N and (*g*) C⋯C contacts.

**Figure 10:**
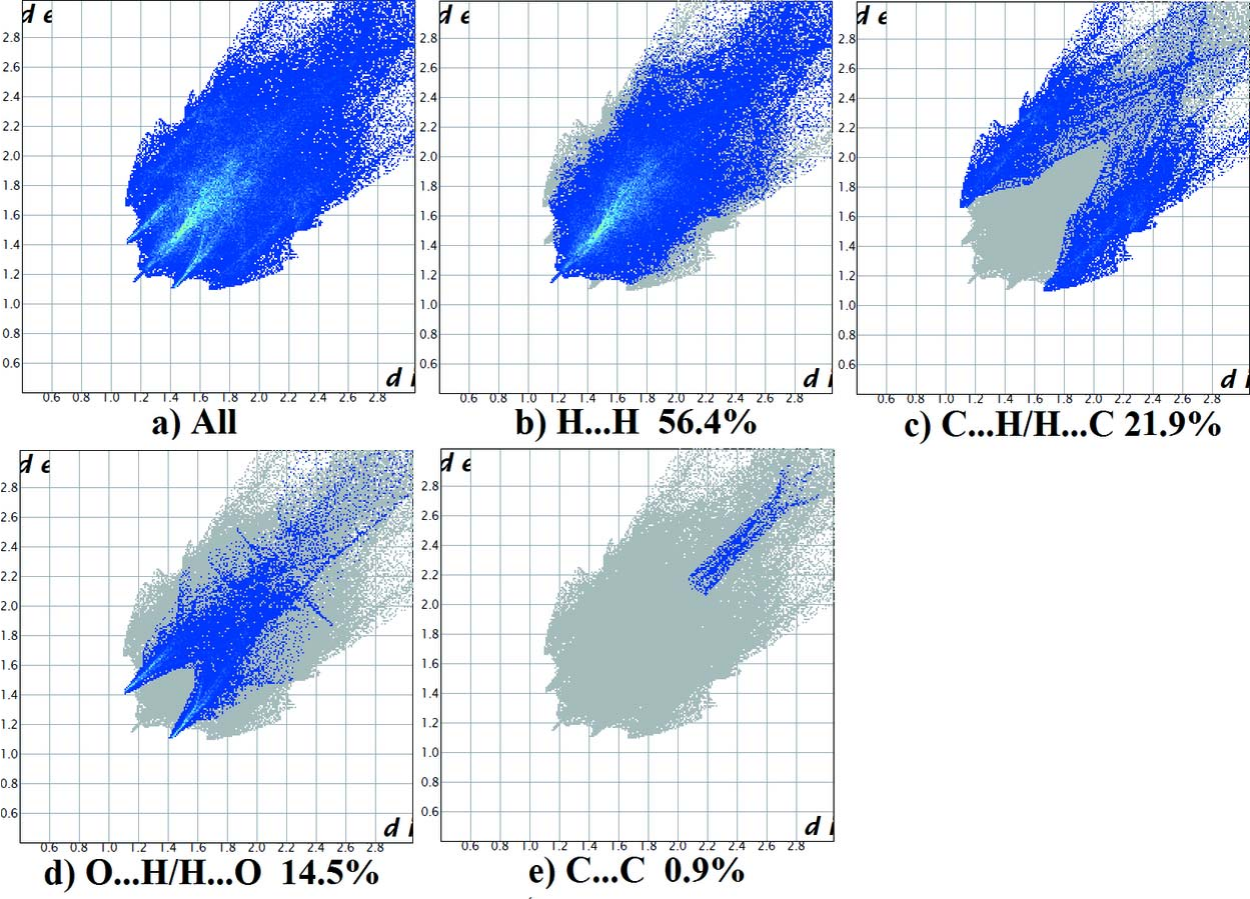
The full two-dimensional fingerprint plot for (II) (*a*), and the fingerprint plots delineated into (*b*) H⋯H, (*c*) C⋯H/H⋯C, (*d*) O⋯H/H⋯O and (*e*) C⋯C contacts.

The fingerprint plots for the two compounds are given in Figs. 9 ▸ and 10 ▸. For (I), they reveal that the principal inter­molecular contacts are H⋯H (44.9%, Fig. 9 ▸ *b*), C⋯H/H⋯C (25.0%, Fig. 9 ▸ *c*), O⋯H/H⋯O (11.8%, Fig. 9 ▸ *d*), S⋯H/H⋯S (5.4%, Fig. 9 ▸ *e*) and N⋯H/H⋯N (4.0%, Fig. 9 ▸ *f*), followed by the C⋯C contacts (3.5%, Fig. 9 ▸ *g*). For (II), they reveal a similar trend, with the principal inter­molecular contacts being H⋯H (56.4%, Fig. 10 ▸ *b*), C⋯H/H⋯C (21.9%, Fig. 10 ▸ *c*), O⋯H/H⋯O (14.5%, Fig. 10 ▸ *d*), followed by the C⋯C contacts (0.9%, Fig. 10 ▸ *e*). In both compounds the H⋯H inter­molecular contacts predominate.

## Database survey   

A search of the Cambridge Structural Database (CSD, Version 5.39, August 2018; Groom *et al.*, 2016 ▸) for the 6′-(4-phen­yl)-6a′-hexa­hydro-2*H*,6′*H*,6b′*H-*spiro­[benzo­pyrano[3,4-*a*]indolizin]-2-one skeleton yielded five hits: namely 6-(4- meth­oxy­phen­yl)-6a-nitro-6,6a,6b,7,8,9,10,12a-octa­hydro­spiro-[chromeno[3,4-*a*]indolizine-12,3-indolin]-2-one (AFONEQ; Devi *et al.*, 2013*a* ▸) and 6-(4-meth­oxy­phen­yl)-6a-nitro-6,6a,6b,7,8,9,10,12a-octa­hydro­spiro­[chromeno[3,4-*a*]indolizine-12,3-indolin]-2-one (FIDCOM; Devi *et al.*, 2013*b* ▸). In addition, the crystal structures of 6-(naphthalen-1-yl)-6a-nitro-6,6a,6b,7,9,11a-hexa­hydro­spiro­[chromeno[3′,4′:3,4]pyrrolo [1,2-*c*]thia­zole-11,11′-indeno­[1,2-*b*]quinoxaline] (XITKUJ and XITKOD; Syed Abuthahir *et al.*, 2019*a* ▸) and 6′-(naphthalen-1-yl)-6a′-nitro-6′,6a′,6b′,7′,8′,9′,10′,12a′-octa­hydro-2*H*-spiro­[ace­naphthyl­ene-1,12′-chromeno[3,4-*a*]indoliz­in]-2-one (XIWRUT01; Syed Abuthahir *et al.*, 2019*b* ▸) were recently reported by some of us. The bond lengths and bond angles are very similar to those reported here for the title compounds.

## Synthesis and crystallization   

**Compound (I):** to a solution of indeno­quinoxalinone (0.232 g, 1.0 mmol) and thia­zolidine-4-carb­oxy­lic acid (0.199 g, 1.5 mmol) in dry toluene, 0.302 g (1.0 mmol) of 2-(naphthalen-1-yl)-3-nitro-2*H*-chromene were added under a nitro­gen atmosphere.

**Compound (II):** to a solution of ace­naphtho­quinone (0.182 g, 1.0 mmol) and pipacolinic acid (0.193 g, 1.5 mmol) in dry toluene, (0.302 g, 1 mmol) of 2-(naphthalen-1-yl)-3-nitro-2*H*-chromene were added under a nitro­gen atmosphere.

The solutions were refluxed for 18 h in a Dean–Stark apparatus to give the cyclo­adducts. After completion of the reactions as indicated by TLC, the solvent was evaporated under reduced pressure. The crude products obtained were purified by column chromatography using hexa­ne/EtOAc (7:3) as eluent (yield 84%). Colourless block-like crystals of the title compounds, suitable for X-ray diffraction analysis, were obtained by slow evaporation of solutions in ethanol.

## Refinement   

Crystal data, data collection and structure refinement details are summarized in Table 3 ▸. All H atoms were positioned geometrically, with N—H = 0.86 Å, C—H = 0.93-0.97 Å, and constrained to ride on their parent atoms with *U*_iso_(H) = 1.5*U*_eq_(C-meth­yl) and 1.2*U*_eq_(N, C) for all other H atoms.

## Supplementary Material

Crystal structure: contains datablock(s) global, I, II. DOI: 10.1107/S205698901901291X/xi2018sup1.cif

Structure factors: contains datablock(s) I. DOI: 10.1107/S205698901901291X/xi2018Isup2.hkl

Structure factors: contains datablock(s) II. DOI: 10.1107/S205698901901291X/xi2018IIsup3.hkl

Supporting information file. DOI: 10.1107/S205698901901291X/xi2018Isup4.cml

Supporting information file. DOI: 10.1107/S205698901901291X/xi2018IIsup5.cml

Additional supporting information:  crystallographic information; 3D view; checkCIF report

## Figures and Tables

**Figure 6:**
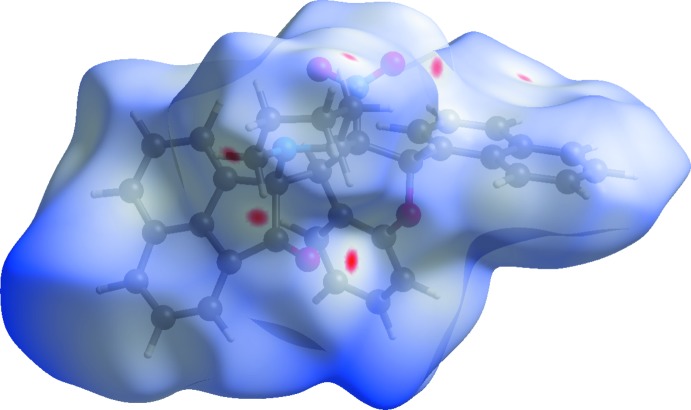
The Hirshfeld surface mapped over *d*_norm_ for (II) mapped over an arbitrary colour scale of −0.080 (red) to 3.098 (blue).

**Table 1:** Hydrogen-bond geometry (Å, °) for (I) *Cg*1 is the centroid of the C9–C14 ring.

*D*—H⋯*A*	*D*—H	H⋯*A*	*D*⋯*A*	*D*—H⋯*A*
C19—H19⋯S1^i^	0.93	2.78	3.640 (3)	156
C23—H23⋯N1	0.98	2.41	3.267 (3)	145
C27—H27*B*⋯O2^ii^	0.97	2.59	3.393 (3)	140
C30—H30⋯O3^iii^	0.93	2.57	3.480 (3)	166
C33—H33⋯O3^iv^	0.93	2.58	3.274 (3)	131
C20—H20⋯*Cg*1^v^	0.93	2.81	3.706 (3)	163

Symmetry codes: (i) 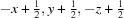; (ii) 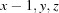; (iii) 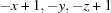; (iv) 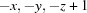; (v) 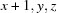.

**Table 2:** Hydrogen-bond geometry (Å, °) for (II) *Cg*1 is the centroid of the C6–C11 ring.

*D*—H⋯*A*	*D*—H	H⋯*A*	*D*⋯*A*	*D*—H⋯*A*
C13—H13*A*⋯O3^i^	0.97	2.59	3.413 (3)	143
C17—H17⋯O1	0.98	2.50	3.148 (2)	124
C32—H32⋯O1^ii^	0.93	2.59	3.318 (3)	135
C35—H35⋯*Cg*1^iii^	0.93	2.92	3.849 (2)	176

Symmetry codes: (i) 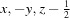; (ii) 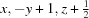; (iii) 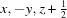.

**Table 3:** Experimental details

	(I)	(II)
Crystal data		
Chemical formula	C_37_H_26_N_4_O_3_S	C_36_H_28_N_2_O_4_
*M*_r_	606.68	552.60
Crystal system, space group	Monoclinic, *P*2_1_/*n*	Monoclinic, *C*2/*c*
Temperature (K)	293	293
*a*, *b*, *c* (Å)	8.3690 (3), 13.2440 (4), 29.2210 (5)	35.7360 (5), 11.4510 (4), 15.3130 (3)
β (°)	93.280 (2)	98.378 (2)
*V* (Å^3^)	3233.52 (16)	6199.4 (3)
*Z*	4	8
Radiation type	Mo *K*α	Mo *K*α
μ (mm^−1^)	0.14	0.08
Crystal size (mm)	0.25 × 0.20 × 0.15	0.30 × 0.24 × 0.22

Data collection		
Diffractometer	Bruker Kappa APEXII CCD	Bruker Kappa APEXII CCD
Absorption correction	Multi-scan (*SADABS*; Bruker, 2008 ▸)	Multi-scan (*SADABS*; Bruker, 2008 ▸)
*T*_min_, *T*_max_	0.741, 0.852	0.742, 0.863
No. of measured, independent and observed [*I* > 2σ(*I*)] reflections	30398, 8083, 4209	24488, 5464, 4002
*R*_int_	0.061	0.027
(sin θ/λ)_max_ (Å^−1^)	0.673	0.595

Refinement		
*R*[*F* ^2^ > 2σ(*F* ^2^)], *wR*(*F* ^2^), *S*	0.057, 0.164, 1.06	0.043, 0.126, 1.07
No. of reflections	8083	5464
No. of parameters	406	380
No. of restraints	1	0
H-atom treatment	H-atom parameters constrained	H-atom parameters constrained
Δρ_max_, Δρ_min_ (e Å^−3^)	0.36, −0.34	0.15, −0.16

Computer programs: *APEX2* and *SAINT* (Bruker, 2008 ▸), *SHELXT2018* (Sheldrick, 2015*a* ▸), *SHELXL2018* (Sheldrick, 2015*b* ▸), *ORTEP-3 for Windows* and *WinGX* (Farrugia, 2012 ▸), *Mercury* (Macrae *et al.*, 2008 ▸), *publCIF* (Westrip, 2010 ▸) and *PLATON* (Spek, 2009 ▸).

## supplementary crystallographic information

### 6-(Naphthalen-1-yl)-6a-nitro-6,6a,6b,7,9,11a-hexahydrospiro[chromeno[3',4':3,4]pyrrolo[1,2-c]thiazole-11,11'-indeno[1,2-b]quinoxaline] (I) Crystal data

**Table:**

C_37_H_26_N_4_O_3_S	*F*(000) = 1264
*M**_r_* = 606.68	*D*_x_ = 1.246 Mg m^−^^3^
Monoclinic, *P*2_1_/*n*	Mo *K*α radiation, λ = 0.71073 Å
*a* = 8.3690 (3) Å	Cell parameters from 8083 reflections
*b* = 13.2440 (4) Å	θ = 1.8–26.9°
*c* = 29.2210 (5) Å	µ = 0.14 mm^−^^1^
β = 93.280 (2)°	*T* = 293 K
*V* = 3233.52 (16) Å^3^	Block, colourless
*Z* = 4	0.25 × 0.20 × 0.15 mm

### 6-(Naphthalen-1-yl)-6a-nitro-6,6a,6b,7,9,11a-hexahydrospiro[chromeno[3',4':3,4]pyrrolo[1,2-c]thiazole-11,11'-indeno[1,2-b]quinoxaline] (I) Data collection

**Table:**

Bruker Kappa APEXII CCD diffractometer	4209 reflections with *I* > 2σ(*I*)
ω and φ scans	*R*_int_ = 0.061
Absorption correction: multi-scan (*SADABS*; Bruker, 2008)	θ_max_ = 28.6°, θ_min_ = 1.4°
*T*_min_ = 0.741, *T*_max_ = 0.852	*h* = −11→10
30398 measured reflections	*k* = −14→17
8083 independent reflections	*l* = −39→39

### 6-(Naphthalen-1-yl)-6a-nitro-6,6a,6b,7,9,11a-hexahydrospiro[chromeno[3',4':3,4]pyrrolo[1,2-c]thiazole-11,11'-indeno[1,2-b]quinoxaline] (I) Refinement

**Table:**

Refinement on *F*^2^	1 restraint
Least-squares matrix: full	Hydrogen site location: inferred from neighbouring sites
*R*[*F*^2^ > 2σ(*F*^2^)] = 0.057	H-atom parameters constrained
*wR*(*F*^2^) = 0.164	*w* = 1/[σ^2^(*F*_o_^2^) + (0.0738*P*)^2^] where *P* = (*F*_o_^2^ + 2*F*_c_^2^)/3
*S* = 1.06	(Δ/σ)_max_ < 0.001
8083 reflections	Δρ_max_ = 0.36 e Å^−^^3^
406 parameters	Δρ_min_ = −0.34 e Å^−^^3^

### 6-(Naphthalen-1-yl)-6a-nitro-6,6a,6b,7,9,11a-hexahydrospiro[chromeno[3',4':3,4]pyrrolo[1,2-c]thiazole-11,11'-indeno[1,2-b]quinoxaline] (I) Special details

**Table:**

Geometry. All esds (except the esd in the dihedral angle between two l.s. planes) are estimated using the full covariance matrix. The cell esds are taken into account individually in the estimation of esds in distances, angles and torsion angles; correlations between esds in cell parameters are only used when they are defined by crystal symmetry. An approximate (isotropic) treatment of cell esds is used for estimating esds involving l.s. planes.

### 6-(Naphthalen-1-yl)-6a-nitro-6,6a,6b,7,9,11a-hexahydrospiro[chromeno[3',4':3,4]pyrrolo[1,2-c]thiazole-11,11'-indeno[1,2-b]quinoxaline] (I) Fractional atomic coordinates and isotropic or equivalent isotropic displacement parameters (Å^2^)

**Table:**

	*x*	*y*	*z*	*U*_iso_*/*U*_eq_	
C1	0.1161 (2)	0.24359 (15)	0.30919 (6)	0.0494 (5)	
C2	0.0918 (2)	0.23613 (16)	0.25673 (7)	0.0541 (5)	
C3	0.1107 (3)	0.15404 (18)	0.22850 (7)	0.0671 (6)	
H3	0.139426	0.091580	0.240969	0.081*	
C4	0.0865 (3)	0.1652 (2)	0.18134 (8)	0.0849 (8)	
H4	0.098128	0.109992	0.162138	0.102*	
C5	0.0448 (3)	0.2591 (3)	0.16285 (9)	0.0936 (9)	
H5	0.031066	0.266237	0.131206	0.112*	
C6	0.0239 (3)	0.3398 (2)	0.18991 (9)	0.0859 (8)	
H6	−0.007778	0.401526	0.177193	0.103*	
C7	0.0505 (2)	0.32971 (17)	0.23738 (7)	0.0614 (6)	
C8	0.0423 (2)	0.40471 (18)	0.27342 (8)	0.0610 (6)	
C9	0.0036 (3)	0.55087 (19)	0.31022 (12)	0.0825 (8)	
C10	−0.0465 (4)	0.6527 (2)	0.31060 (17)	0.1145 (11)	
H10	−0.076570	0.684749	0.283137	0.137*	
C11	−0.0510 (5)	0.7035 (3)	0.3502 (2)	0.1415 (17)	
H11	−0.084652	0.770462	0.349812	0.170*	
C12	−0.0066 (5)	0.6582 (3)	0.3912 (2)	0.1382 (15)	
H12	−0.010154	0.695207	0.418145	0.166*	
C13	0.0430 (4)	0.5595 (2)	0.39324 (12)	0.1058 (10)	
H13	0.073851	0.529893	0.421227	0.127*	
C14	0.0463 (3)	0.50397 (19)	0.35256 (10)	0.0750 (7)	
C15	0.0833 (2)	0.35657 (16)	0.31577 (8)	0.0541 (5)	
C16	0.2865 (2)	0.20774 (14)	0.32709 (6)	0.0472 (5)	
H16	0.325781	0.161182	0.304208	0.057*	
C17	0.4111 (2)	0.28888 (15)	0.33502 (7)	0.0517 (5)	
C18	0.4752 (2)	0.33919 (18)	0.29859 (8)	0.0670 (6)	
H18	0.439990	0.323138	0.268711	0.080*	
C19	0.5897 (3)	0.4123 (2)	0.30622 (9)	0.0801 (7)	
H19	0.631402	0.445264	0.281466	0.096*	
C20	0.6434 (3)	0.4371 (2)	0.35007 (10)	0.0846 (8)	
H20	0.720422	0.487177	0.355019	0.102*	
C21	0.5830 (3)	0.38786 (18)	0.38640 (9)	0.0722 (7)	
H21	0.619996	0.403698	0.416151	0.087*	
C22	0.4672 (2)	0.31471 (16)	0.37893 (7)	0.0556 (5)	
C23	0.2641 (2)	0.21920 (14)	0.41284 (6)	0.0472 (5)	
H23	0.182414	0.271326	0.406921	0.057*	
C24	0.25919 (19)	0.14506 (12)	0.37099 (6)	0.0446 (4)	
C25	0.0895 (2)	0.10286 (14)	0.36166 (6)	0.0482 (5)	
H25	0.040553	0.091076	0.390862	0.058*	
C26	0.0710 (3)	0.00792 (16)	0.33174 (7)	0.0591 (5)	
H26A	0.088186	−0.052686	0.350003	0.071*	
H26B	0.146009	0.008585	0.307642	0.071*	
C27	−0.1468 (2)	0.15173 (17)	0.31845 (8)	0.0665 (6)	
H27A	−0.174584	0.186668	0.289926	0.080*	
H27B	−0.228995	0.165400	0.339706	0.080*	
C28	0.2370 (2)	0.17076 (14)	0.45860 (6)	0.0466 (5)	
C29	0.3636 (3)	0.12777 (16)	0.48297 (7)	0.0579 (5)	
H29	0.463434	0.127450	0.470563	0.069*	
C30	0.3470 (3)	0.08386 (17)	0.52642 (7)	0.0670 (6)	
H30	0.435212	0.056120	0.542655	0.080*	
C31	0.2015 (3)	0.08258 (16)	0.54417 (7)	0.0621 (6)	
H31	0.190245	0.052815	0.572633	0.074*	
C32	0.0676 (2)	0.12492 (14)	0.52078 (6)	0.0523 (5)	
C33	−0.0829 (3)	0.12485 (18)	0.53942 (7)	0.0656 (6)	
H33	−0.094090	0.094106	0.567672	0.079*	
C34	−0.2109 (3)	0.1675 (2)	0.51796 (8)	0.0789 (7)	
H34	−0.309462	0.165333	0.531071	0.095*	
C35	−0.1964 (3)	0.21564 (19)	0.47545 (8)	0.0747 (7)	
H35	−0.285147	0.246448	0.460805	0.090*	
C36	−0.0535 (2)	0.21727 (16)	0.45578 (7)	0.0582 (5)	
H36	−0.045876	0.249053	0.427606	0.070*	
C37	0.0839 (2)	0.17177 (13)	0.47715 (6)	0.0460 (5)	
N1	0.0872 (2)	0.40293 (14)	0.35511 (7)	0.0660 (5)	
N2	0.0036 (2)	0.49945 (16)	0.26964 (8)	0.0773 (6)	
N3	0.00650 (17)	0.18572 (12)	0.33735 (5)	0.0503 (4)	
N4	0.37670 (19)	0.06748 (12)	0.37895 (5)	0.0512 (4)	
O1	0.41679 (16)	0.26684 (11)	0.41708 (4)	0.0619 (4)	
O2	0.51538 (18)	0.08236 (11)	0.36905 (6)	0.0754 (5)	
O3	0.33881 (17)	−0.01166 (11)	0.39739 (5)	0.0637 (4)	
S1	−0.13165 (8)	0.01440 (5)	0.30819 (2)	0.0791 (2)	

### 6-(Naphthalen-1-yl)-6a-nitro-6,6a,6b,7,9,11a-hexahydrospiro[chromeno[3',4':3,4]pyrrolo[1,2-c]thiazole-11,11'-indeno[1,2-b]quinoxaline] (I) Atomic displacement parameters (Å^2^)

**Table:**

	*U*^11^	*U*^22^	*U*^33^	*U*^12^	*U*^13^	*U*^23^
C1	0.0494 (11)	0.0584 (13)	0.0412 (10)	−0.0039 (9)	0.0084 (9)	0.0061 (9)
C2	0.0503 (12)	0.0696 (14)	0.0425 (11)	−0.0071 (10)	0.0034 (9)	0.0093 (10)
C3	0.0787 (15)	0.0808 (16)	0.0424 (12)	−0.0018 (12)	0.0073 (11)	0.0045 (11)
C4	0.0974 (19)	0.110 (2)	0.0471 (14)	−0.0118 (16)	0.0034 (13)	−0.0004 (14)
C5	0.113 (2)	0.125 (3)	0.0428 (14)	−0.0156 (18)	0.0011 (14)	0.0160 (17)
C6	0.0889 (19)	0.106 (2)	0.0622 (17)	−0.0077 (15)	−0.0052 (14)	0.0348 (16)
C7	0.0522 (13)	0.0786 (16)	0.0534 (13)	−0.0104 (11)	0.0015 (10)	0.0171 (12)
C8	0.0456 (12)	0.0651 (16)	0.0719 (15)	−0.0070 (10)	0.0005 (10)	0.0185 (12)
C9	0.0619 (16)	0.0558 (17)	0.130 (3)	−0.0140 (12)	0.0078 (16)	0.0102 (17)
C10	0.100 (2)	0.059 (2)	0.184 (4)	−0.0076 (16)	0.007 (2)	0.015 (2)
C11	0.134 (3)	0.056 (2)	0.235 (6)	−0.007 (2)	0.020 (3)	−0.013 (3)
C12	0.135 (3)	0.079 (3)	0.201 (5)	−0.013 (2)	0.019 (3)	−0.057 (3)
C13	0.113 (2)	0.075 (2)	0.129 (3)	−0.0083 (16)	0.0026 (19)	−0.0351 (18)
C14	0.0578 (15)	0.0589 (17)	0.109 (2)	−0.0086 (11)	0.0058 (13)	−0.0062 (15)
C15	0.0417 (11)	0.0601 (14)	0.0606 (13)	−0.0059 (9)	0.0040 (9)	0.0053 (11)
C16	0.0443 (11)	0.0593 (12)	0.0388 (10)	−0.0001 (9)	0.0087 (8)	0.0067 (9)
C17	0.0411 (11)	0.0617 (13)	0.0528 (12)	−0.0031 (9)	0.0087 (9)	0.0111 (10)
C18	0.0544 (13)	0.0859 (16)	0.0617 (14)	−0.0054 (12)	0.0137 (10)	0.0237 (12)
C19	0.0638 (15)	0.100 (2)	0.0775 (18)	−0.0176 (14)	0.0161 (13)	0.0349 (14)
C20	0.0656 (16)	0.0879 (19)	0.101 (2)	−0.0264 (13)	0.0080 (14)	0.0270 (15)
C21	0.0594 (14)	0.0815 (16)	0.0751 (16)	−0.0216 (12)	−0.0003 (12)	0.0125 (12)
C22	0.0478 (11)	0.0656 (14)	0.0536 (13)	−0.0129 (10)	0.0062 (9)	0.0096 (10)
C23	0.0471 (11)	0.0532 (12)	0.0414 (10)	−0.0103 (9)	0.0039 (8)	0.0013 (8)
C24	0.0446 (11)	0.0522 (11)	0.0374 (10)	−0.0006 (9)	0.0064 (8)	0.0052 (8)
C25	0.0511 (11)	0.0581 (12)	0.0357 (10)	−0.0109 (9)	0.0063 (8)	0.0007 (9)
C26	0.0691 (14)	0.0637 (14)	0.0451 (11)	−0.0129 (10)	0.0079 (10)	−0.0040 (9)
C27	0.0500 (13)	0.0894 (17)	0.0599 (14)	−0.0130 (11)	0.0024 (10)	0.0090 (11)
C28	0.0513 (12)	0.0513 (11)	0.0372 (10)	−0.0065 (9)	0.0027 (9)	−0.0014 (8)
C29	0.0536 (12)	0.0751 (14)	0.0451 (11)	−0.0008 (11)	0.0038 (10)	0.0002 (10)
C30	0.0722 (16)	0.0801 (16)	0.0479 (12)	0.0037 (12)	−0.0027 (11)	0.0071 (11)
C31	0.0766 (16)	0.0703 (15)	0.0394 (11)	−0.0079 (12)	0.0037 (11)	0.0063 (10)
C32	0.0638 (13)	0.0558 (12)	0.0376 (10)	−0.0159 (10)	0.0047 (10)	−0.0043 (9)
C33	0.0643 (15)	0.0885 (17)	0.0446 (12)	−0.0236 (12)	0.0082 (11)	−0.0002 (11)
C34	0.0611 (15)	0.118 (2)	0.0595 (15)	−0.0158 (14)	0.0195 (12)	−0.0035 (14)
C35	0.0548 (14)	0.1079 (19)	0.0618 (15)	0.0005 (12)	0.0062 (11)	0.0050 (13)
C36	0.0548 (13)	0.0741 (14)	0.0461 (11)	−0.0036 (11)	0.0069 (10)	0.0040 (10)
C37	0.0506 (11)	0.0487 (11)	0.0389 (10)	−0.0108 (9)	0.0036 (9)	−0.0045 (8)
N1	0.0608 (11)	0.0621 (13)	0.0754 (14)	−0.0053 (9)	0.0074 (10)	−0.0065 (10)
N2	0.0665 (13)	0.0621 (14)	0.1031 (18)	−0.0071 (10)	0.0031 (11)	0.0214 (12)
N3	0.0424 (9)	0.0652 (11)	0.0437 (9)	−0.0082 (8)	0.0065 (7)	0.0062 (8)
N4	0.0532 (11)	0.0584 (11)	0.0425 (9)	−0.0018 (9)	0.0063 (8)	0.0021 (8)
O1	0.0589 (9)	0.0785 (10)	0.0483 (8)	−0.0286 (7)	0.0026 (6)	0.0055 (7)
O2	0.0502 (9)	0.0822 (11)	0.0953 (12)	0.0063 (7)	0.0185 (8)	0.0192 (9)
O3	0.0709 (10)	0.0622 (9)	0.0586 (9)	−0.0008 (7)	0.0085 (7)	0.0144 (7)
S1	0.0740 (4)	0.0978 (5)	0.0647 (4)	−0.0289 (3)	−0.0022 (3)	−0.0161 (3)

### 6-(Naphthalen-1-yl)-6a-nitro-6,6a,6b,7,9,11a-hexahydrospiro[chromeno[3',4':3,4]pyrrolo[1,2-c]thiazole-11,11'-indeno[1,2-b]quinoxaline] (I) Geometric parameters (Å, º)

**Table:**

C1—N3	1.481 (2)	C20—H20	0.9300
C1—C15	1.535 (3)	C21—C22	1.379 (3)
C1—C2	1.538 (3)	C21—H21	0.9300
C1—C16	1.564 (3)	C22—O1	1.369 (2)
C2—C3	1.379 (3)	C23—O1	1.424 (2)
C2—C7	1.397 (3)	C23—C28	1.512 (2)
C3—C4	1.389 (3)	C23—C24	1.567 (2)
C3—H3	0.9300	C23—H23	0.9800
C4—C5	1.393 (4)	C24—N4	1.4321 (9)
C4—H4	0.9300	C24—C25	1.536 (2)
C5—C6	1.347 (4)	C25—N3	1.461 (2)
C5—H5	0.9300	C25—C26	1.534 (3)
C6—C7	1.399 (3)	C25—H25	0.9800
C6—H6	0.9300	C26—S1	1.795 (2)
C7—C8	1.452 (3)	C26—H26A	0.9700
C8—N2	1.299 (3)	C26—H26B	0.9700
C8—C15	1.417 (3)	C27—N3	1.439 (2)
C9—N2	1.367 (3)	C27—S1	1.849 (2)
C9—C10	1.412 (4)	C27—H27A	0.9700
C9—C14	1.412 (4)	C27—H27B	0.9700
C10—C11	1.340 (6)	C28—C29	1.366 (3)
C10—H10	0.9300	C28—C37	1.420 (3)
C11—C12	1.373 (6)	C29—C30	1.411 (3)
C11—H11	0.9300	C29—H29	0.9300
C12—C13	1.372 (5)	C30—C31	1.351 (3)
C12—H12	0.9300	C30—H30	0.9300
C13—C14	1.399 (4)	C31—C32	1.396 (3)
C13—H13	0.9300	C31—H31	0.9300
C14—N1	1.382 (3)	C32—C33	1.401 (3)
C15—N1	1.302 (3)	C32—C37	1.431 (3)
C16—C17	1.506 (3)	C33—C34	1.335 (3)
C16—C24	1.556 (2)	C33—H33	0.9300
C16—H16	0.9800	C34—C35	1.408 (3)
C17—C22	1.384 (3)	C34—H34	0.9300
C17—C18	1.389 (3)	C35—C36	1.356 (3)
C18—C19	1.371 (3)	C35—H35	0.9300
C18—H18	0.9300	C36—C37	1.412 (3)
C19—C20	1.374 (3)	C36—H36	0.9300
C19—H19	0.9300	N4—O2	1.2279 (19)
C20—C21	1.368 (3)	N4—O3	1.2285 (19)
			
N3—C1—C15	108.30 (15)	C21—C22—C17	121.22 (19)
N3—C1—C2	118.02 (15)	O1—C23—C28	106.87 (14)
C15—C1—C2	99.97 (15)	O1—C23—C24	109.20 (14)
N3—C1—C16	103.82 (14)	C28—C23—C24	115.20 (15)
C15—C1—C16	114.83 (15)	O1—C23—H23	108.5
C2—C1—C16	112.33 (15)	C28—C23—H23	108.5
C3—C2—C7	119.43 (19)	C24—C23—H23	108.5
C3—C2—C1	129.31 (19)	N4—C24—C25	112.77 (15)
C7—C2—C1	111.21 (18)	N4—C24—C16	112.63 (14)
C2—C3—C4	119.6 (2)	C25—C24—C16	103.05 (13)
C2—C3—H3	120.2	N4—C24—C23	109.61 (14)
C4—C3—H3	120.2	C25—C24—C23	110.44 (13)
C3—C4—C5	119.9 (2)	C16—C24—C23	108.10 (14)
C3—C4—H4	120.0	N3—C25—C26	107.98 (15)
C5—C4—H4	120.0	N3—C25—C24	102.83 (13)
C6—C5—C4	121.3 (2)	C26—C25—C24	117.50 (16)
C6—C5—H5	119.4	N3—C25—H25	109.4
C4—C5—H5	119.4	C26—C25—H25	109.4
C5—C6—C7	119.2 (2)	C24—C25—H25	109.4
C5—C6—H6	120.4	C25—C26—S1	104.00 (14)
C7—C6—H6	120.4	C25—C26—H26A	111.0
C2—C7—C6	120.5 (2)	S1—C26—H26A	111.0
C2—C7—C8	109.52 (19)	C25—C26—H26B	111.0
C6—C7—C8	130.0 (2)	S1—C26—H26B	111.0
N2—C8—C15	123.8 (2)	H26A—C26—H26B	109.0
N2—C8—C7	128.4 (2)	N3—C27—S1	107.58 (14)
C15—C8—C7	107.9 (2)	N3—C27—H27A	110.2
N2—C9—C10	119.8 (3)	S1—C27—H27A	110.2
N2—C9—C14	121.9 (2)	N3—C27—H27B	110.2
C10—C9—C14	118.3 (3)	S1—C27—H27B	110.2
C11—C10—C9	120.6 (4)	H27A—C27—H27B	108.5
C11—C10—H10	119.7	C29—C28—C37	119.65 (17)
C9—C10—H10	119.7	C29—C28—C23	119.15 (17)
C10—C11—C12	120.9 (4)	C37—C28—C23	121.18 (16)
C10—C11—H11	119.5	C28—C29—C30	121.9 (2)
C12—C11—H11	119.5	C28—C29—H29	119.1
C13—C12—C11	121.4 (4)	C30—C29—H29	119.1
C13—C12—H12	119.3	C31—C30—C29	119.2 (2)
C11—C12—H12	119.3	C31—C30—H30	120.4
C12—C13—C14	118.9 (4)	C29—C30—H30	120.4
C12—C13—H13	120.5	C30—C31—C32	121.53 (19)
C14—C13—H13	120.5	C30—C31—H31	119.2
N1—C14—C13	118.7 (3)	C32—C31—H31	119.2
N1—C14—C9	121.5 (2)	C31—C32—C33	121.59 (19)
C13—C14—C9	119.8 (3)	C31—C32—C37	119.71 (18)
N1—C15—C8	123.5 (2)	C33—C32—C37	118.69 (19)
N1—C15—C1	125.07 (19)	C34—C33—C32	122.1 (2)
C8—C15—C1	111.42 (19)	C34—C33—H33	118.9
C17—C16—C24	112.77 (15)	C32—C33—H33	118.9
C17—C16—C1	116.40 (16)	C33—C34—C35	119.9 (2)
C24—C16—C1	105.28 (13)	C33—C34—H34	120.0
C17—C16—H16	107.3	C35—C34—H34	120.0
C24—C16—H16	107.3	C36—C35—C34	120.2 (2)
C1—C16—H16	107.3	C36—C35—H35	119.9
C22—C17—C18	117.82 (19)	C34—C35—H35	119.9
C22—C17—C16	120.94 (16)	C35—C36—C37	121.41 (19)
C18—C17—C16	121.24 (19)	C35—C36—H36	119.3
C19—C18—C17	120.7 (2)	C37—C36—H36	119.3
C19—C18—H18	119.6	C36—C37—C28	124.43 (17)
C17—C18—H18	119.6	C36—C37—C32	117.55 (17)
C18—C19—C20	120.6 (2)	C28—C37—C32	118.01 (17)
C18—C19—H19	119.7	C15—N1—C14	114.5 (2)
C20—C19—H19	119.7	C8—N2—C9	114.8 (2)
C21—C20—C19	119.6 (2)	C27—N3—C25	109.89 (15)
C21—C20—H20	120.2	C27—N3—C1	121.03 (15)
C19—C20—H20	120.2	C25—N3—C1	111.50 (14)
C20—C21—C22	120.0 (2)	O2—N4—O3	120.76 (14)
C20—C21—H21	120.0	O2—N4—C24	119.74 (16)
C22—C21—H21	120.0	O3—N4—C24	119.43 (15)
O1—C22—C21	116.28 (19)	C22—O1—C23	116.79 (14)
O1—C22—C17	122.44 (17)	C26—S1—C27	93.26 (9)
			
N3—C1—C2—C3	−66.0 (3)	C28—C23—C24—N4	−57.8 (2)
C15—C1—C2—C3	176.9 (2)	O1—C23—C24—C25	−172.68 (14)
C16—C1—C2—C3	54.7 (3)	C28—C23—C24—C25	67.09 (19)
N3—C1—C2—C7	116.51 (19)	O1—C23—C24—C16	−60.63 (17)
C15—C1—C2—C7	−0.53 (19)	C28—C23—C24—C16	179.14 (14)
C16—C1—C2—C7	−122.75 (17)	N4—C24—C25—N3	−158.22 (14)
C7—C2—C3—C4	−0.8 (3)	C16—C24—C25—N3	−36.51 (16)
C1—C2—C3—C4	−178.1 (2)	C23—C24—C25—N3	78.75 (16)
C2—C3—C4—C5	0.6 (4)	N4—C24—C25—C26	−39.8 (2)
C3—C4—C5—C6	−1.4 (4)	C16—C24—C25—C26	81.88 (18)
C4—C5—C6—C7	2.4 (4)	C23—C24—C25—C26	−162.86 (16)
C3—C2—C7—C6	1.8 (3)	N3—C25—C26—S1	−39.77 (16)
C1—C2—C7—C6	179.58 (19)	C24—C25—C26—S1	−155.37 (13)
C3—C2—C7—C8	−178.23 (18)	O1—C23—C28—C29	−37.1 (2)
C1—C2—C7—C8	−0.5 (2)	C24—C23—C28—C29	84.4 (2)
C5—C6—C7—C2	−2.6 (3)	O1—C23—C28—C37	141.13 (16)
C5—C6—C7—C8	177.5 (2)	C24—C23—C28—C37	−97.4 (2)
C2—C7—C8—N2	−177.9 (2)	C37—C28—C29—C30	0.0 (3)
C6—C7—C8—N2	2.0 (4)	C23—C28—C29—C30	178.23 (18)
C2—C7—C8—C15	1.4 (2)	C28—C29—C30—C31	1.2 (3)
C6—C7—C8—C15	−178.7 (2)	C29—C30—C31—C32	−0.9 (3)
N2—C9—C10—C11	178.4 (3)	C30—C31—C32—C33	−179.1 (2)
C14—C9—C10—C11	0.9 (4)	C30—C31—C32—C37	−0.6 (3)
C9—C10—C11—C12	0.3 (6)	C31—C32—C33—C34	178.2 (2)
C10—C11—C12—C13	−0.4 (6)	C37—C32—C33—C34	−0.4 (3)
C11—C12—C13—C14	−0.7 (5)	C32—C33—C34—C35	−0.9 (4)
C12—C13—C14—N1	−176.2 (3)	C33—C34—C35—C36	1.2 (4)
C12—C13—C14—C9	1.8 (4)	C34—C35—C36—C37	−0.4 (3)
N2—C9—C14—N1	−1.4 (3)	C35—C36—C37—C28	−179.9 (2)
C10—C9—C14—N1	176.1 (2)	C35—C36—C37—C32	−0.9 (3)
N2—C9—C14—C13	−179.4 (2)	C29—C28—C37—C36	177.57 (18)
C10—C9—C14—C13	−1.9 (4)	C23—C28—C37—C36	−0.6 (3)
N2—C8—C15—N1	−0.5 (3)	C29—C28—C37—C32	−1.4 (3)
C7—C8—C15—N1	−179.83 (18)	C23—C28—C37—C32	−179.62 (16)
N2—C8—C15—C1	177.54 (18)	C31—C32—C37—C36	−177.34 (18)
C7—C8—C15—C1	−1.8 (2)	C33—C32—C37—C36	1.3 (3)
N3—C1—C15—N1	55.3 (2)	C31—C32—C37—C28	1.7 (3)
C2—C1—C15—N1	179.41 (18)	C33—C32—C37—C28	−179.68 (17)
C16—C1—C15—N1	−60.2 (2)	C8—C15—N1—C14	0.6 (3)
N3—C1—C15—C8	−122.67 (17)	C1—C15—N1—C14	−177.19 (18)
C2—C1—C15—C8	1.42 (19)	C13—C14—N1—C15	178.3 (2)
C16—C1—C15—C8	121.85 (17)	C9—C14—N1—C15	0.3 (3)
N3—C1—C16—C17	−135.76 (16)	C15—C8—N2—C9	−0.5 (3)
C15—C1—C16—C17	−17.7 (2)	C7—C8—N2—C9	178.7 (2)
C2—C1—C16—C17	95.63 (19)	C10—C9—N2—C8	−176.0 (2)
N3—C1—C16—C24	−10.06 (18)	C14—C9—N2—C8	1.4 (3)
C15—C1—C16—C24	107.98 (17)	S1—C27—N3—C25	−27.14 (18)
C2—C1—C16—C24	−138.67 (16)	S1—C27—N3—C1	105.11 (17)
C24—C16—C17—C22	−12.0 (3)	C26—C25—N3—C27	44.43 (19)
C1—C16—C17—C22	109.8 (2)	C24—C25—N3—C27	169.30 (15)
C24—C16—C17—C18	167.06 (17)	C26—C25—N3—C1	−92.60 (17)
C1—C16—C17—C18	−71.1 (2)	C24—C25—N3—C1	32.28 (18)
C22—C17—C18—C19	−0.3 (3)	C15—C1—N3—C27	92.1 (2)
C16—C17—C18—C19	−179.3 (2)	C2—C1—N3—C27	−20.4 (3)
C17—C18—C19—C20	0.0 (4)	C16—C1—N3—C27	−145.47 (17)
C18—C19—C20—C21	0.6 (4)	C15—C1—N3—C25	−136.36 (16)
C19—C20—C21—C22	−0.9 (4)	C2—C1—N3—C25	111.15 (18)
C20—C21—C22—O1	178.0 (2)	C16—C1—N3—C25	−13.90 (19)
C20—C21—C22—C17	0.7 (4)	C25—C24—N4—O2	151.16 (16)
C18—C17—C22—O1	−177.23 (18)	C16—C24—N4—O2	35.0 (2)
C16—C17—C22—O1	1.8 (3)	C23—C24—N4—O2	−85.35 (19)
C18—C17—C22—C21	−0.1 (3)	C25—C24—N4—O3	−31.9 (2)
C16—C17—C22—C21	179.00 (19)	C16—C24—N4—O3	−147.98 (16)
C17—C16—C24—N4	−81.57 (19)	C23—C24—N4—O3	91.63 (18)
C1—C16—C24—N4	150.52 (15)	C21—C22—O1—C23	158.47 (18)
C17—C16—C24—C25	156.62 (15)	C17—C22—O1—C23	−24.2 (3)
C1—C16—C24—C25	28.71 (18)	C28—C23—O1—C22	179.04 (16)
C17—C16—C24—C23	39.69 (19)	C24—C23—O1—C22	53.8 (2)
C1—C16—C24—C23	−88.23 (16)	C25—C26—S1—C27	20.61 (14)
O1—C23—C24—N4	62.48 (18)	N3—C27—S1—C26	2.64 (15)

### 6-(Naphthalen-1-yl)-6a-nitro-6,6a,6b,7,9,11a-hexahydrospiro[chromeno[3',4':3,4]pyrrolo[1,2-c]thiazole-11,11'-indeno[1,2-b]quinoxaline] (I) Hydrogen-bond geometry (Å, º)

*Cg*1 is the centroid of the C9–C14 ring.

**Table:**

*D*—H···*A*	*D*—H	H···*A*	*D*···*A*	*D*—H···*A*
C19—H19···S1^i^	0.93	2.78	3.640 (3)	156
C23—H23···N1	0.98	2.41	3.267 (3)	145
C27—H27*B*···O2^ii^	0.97	2.59	3.393 (3)	140
C30—H30···O3^iii^	0.93	2.57	3.480 (3)	166
C33—H33···O3^iv^	0.93	2.58	3.274 (3)	131
C20—H20···*Cg*1^v^	0.93	2.81	3.706 (3)	163

Symmetry codes: (i) −*x*+1/2, *y*+1/2, −*z*+1/2; (ii) *x*−1, *y*, *z*; (iii) −*x*+1, −*y*, −*z*+1; (iv) −*x*, −*y*, −*z*+1; (v) *x*+1, *y*, *z*.

### 6'-(nNphthalen-1-yl)-6a'-nitro-6',6a',6b',7',8',9',10',12a'-octahydro-2H-spiro[acenaphthylene-1,12'-chromeno[3,4-a]indolizin]-2-one (II) Crystal data

**Table:**

C_36_H_28_N_2_O_4_	*F*(000) = 2320
*M**_r_* = 552.60	*D*_x_ = 1.184 Mg m^−^^3^
Monoclinic, *C*2/*c*	Mo *K*α radiation, λ = 0.71073 Å
*a* = 35.7360 (5) Å	Cell parameters from 5464 reflections
*b* = 11.4510 (4) Å	θ = 1.8–26.9°
*c* = 15.3130 (3) Å	µ = 0.08 mm^−^^1^
β = 98.378 (2)°	*T* = 293 K
*V* = 6199.4 (3) Å^3^	Block, colourless
*Z* = 8	0.30 × 0.24 × 0.22 mm

### 6'-(nNphthalen-1-yl)-6a'-nitro-6',6a',6b',7',8',9',10',12a'-octahydro-2H-spiro[acenaphthylene-1,12'-chromeno[3,4-a]indolizin]-2-one (II) Data collection

**Table:**

Bruker Kappa APEXII CCD diffractometer	4002 reflections with *I* > 2σ(*I*)
ω and φ scans	*R*_int_ = 0.027
Absorption correction: multi-scan (*SADABS*; Bruker, 2008)	θ_max_ = 25.0°, θ_min_ = 1.2°
*T*_min_ = 0.742, *T*_max_ = 0.863	*h* = −38→42
24488 measured reflections	*k* = −12→13
5464 independent reflections	*l* = −18→18

### 6'-(nNphthalen-1-yl)-6a'-nitro-6',6a',6b',7',8',9',10',12a'-octahydro-2H-spiro[acenaphthylene-1,12'-chromeno[3,4-a]indolizin]-2-one (II) Refinement

**Table:**

Refinement on *F*^2^	Hydrogen site location: inferred from neighbouring sites
Least-squares matrix: full	H-atom parameters constrained
*R*[*F*^2^ > 2σ(*F*^2^)] = 0.043	*w* = 1/[σ^2^(*F*_o_^2^) + (0.0521*P*)^2^ + 3.3964*P*] where *P* = (*F*_o_^2^ + 2*F*_c_^2^)/3
*wR*(*F*^2^) = 0.126	(Δ/σ)_max_ < 0.001
*S* = 1.07	Δρ_max_ = 0.15 e Å^−^^3^
5464 reflections	Δρ_min_ = −0.16 e Å^−^^3^
380 parameters	Extinction correction: SHELXL2018/3 (Sheldrick 2015b), Fc^*^=kFc[1+0.001xFc^2^λ^3^/sin(2θ)]^-1/4^
0 restraints	Extinction coefficient: 0.00076 (10)

### 6'-(nNphthalen-1-yl)-6a'-nitro-6',6a',6b',7',8',9',10',12a'-octahydro-2H-spiro[acenaphthylene-1,12'-chromeno[3,4-a]indolizin]-2-one (II) Special details

**Table:**

Geometry. All esds (except the esd in the dihedral angle between two l.s. planes) are estimated using the full covariance matrix. The cell esds are taken into account individually in the estimation of esds in distances, angles and torsion angles; correlations between esds in cell parameters are only used when they are defined by crystal symmetry. An approximate (isotropic) treatment of cell esds is used for estimating esds involving l.s. planes.

### 6'-(nNphthalen-1-yl)-6a'-nitro-6',6a',6b',7',8',9',10',12a'-octahydro-2H-spiro[acenaphthylene-1,12'-chromeno[3,4-a]indolizin]-2-one (II) Fractional atomic coordinates and isotropic or equivalent isotropic displacement parameters (Å^2^)

**Table:**

	*x*	*y*	*z*	*U*_iso_*/*U*_eq_	
C1	0.11638 (5)	0.06508 (18)	0.21020 (13)	0.0526 (5)	
C2	0.08463 (6)	−0.01305 (19)	0.17739 (14)	0.0626 (6)	
C3	0.05810 (7)	−0.0115 (3)	0.10306 (19)	0.0961 (9)	
H3	0.057587	0.048710	0.062178	0.115*	
C4	0.03208 (9)	−0.1019 (4)	0.0903 (3)	0.1207 (12)	
H4	0.013886	−0.101140	0.040225	0.145*	
C5	0.03227 (8)	−0.1916 (3)	0.1486 (3)	0.1065 (11)	
H5	0.014260	−0.250396	0.137433	0.128*	
C6	0.05926 (7)	−0.1975 (2)	0.2259 (2)	0.0786 (7)	
C7	0.06434 (8)	−0.2845 (2)	0.2910 (3)	0.0942 (9)	
H7	0.047848	−0.347774	0.286966	0.113*	
C8	0.09285 (9)	−0.2781 (2)	0.3597 (2)	0.0951 (9)	
H8	0.095799	−0.338284	0.400921	0.114*	
C9	0.11834 (7)	−0.18250 (18)	0.37060 (18)	0.0755 (7)	
H9	0.137421	−0.178846	0.418798	0.091*	
C10	0.11421 (6)	−0.09664 (16)	0.30913 (14)	0.0542 (5)	
C11	0.08490 (5)	−0.10469 (17)	0.23760 (15)	0.0588 (5)	
C12	0.13643 (5)	0.01453 (15)	0.30137 (12)	0.0460 (4)	
C13	0.18850 (6)	−0.06500 (19)	0.22802 (14)	0.0631 (6)	
H13A	0.180208	−0.020888	0.174621	0.076*	
H13B	0.176720	−0.141442	0.221732	0.076*	
C14	0.23091 (6)	−0.0782 (2)	0.24037 (14)	0.0674 (6)	
H14A	0.238852	−0.129872	0.289782	0.081*	
H14B	0.238469	−0.113015	0.187899	0.081*	
C15	0.24999 (6)	0.0384 (2)	0.25728 (14)	0.0629 (6)	
H15A	0.243259	0.088851	0.206529	0.075*	
H15B	0.277228	0.028152	0.266245	0.075*	
C16	0.23775 (5)	0.09456 (17)	0.33870 (12)	0.0507 (5)	
H16A	0.245424	0.045909	0.390051	0.061*	
H16B	0.249717	0.170305	0.349153	0.061*	
C17	0.19517 (5)	0.10841 (14)	0.32397 (11)	0.0405 (4)	
H17	0.188258	0.159896	0.273058	0.049*	
C18	0.17508 (5)	0.15277 (14)	0.40030 (10)	0.0388 (4)	
C19	0.13489 (5)	0.10116 (15)	0.38040 (11)	0.0435 (4)	
H19	0.129935	0.055939	0.431831	0.052*	
C20	0.10467 (5)	0.19219 (17)	0.36118 (12)	0.0487 (5)	
C21	0.06688 (6)	0.1676 (2)	0.36418 (15)	0.0669 (6)	
H21	0.059928	0.094245	0.382387	0.080*	
C22	0.03949 (7)	0.2515 (3)	0.34024 (19)	0.0896 (8)	
H22	0.014138	0.234324	0.341579	0.108*	
C23	0.04983 (8)	0.3603 (3)	0.3145 (2)	0.0908 (8)	
H23	0.031290	0.416307	0.298034	0.109*	
C24	0.08670 (7)	0.3874 (2)	0.31264 (15)	0.0703 (6)	
H24	0.093426	0.461806	0.296162	0.084*	
C25	0.11409 (6)	0.30341 (17)	0.33549 (12)	0.0509 (5)	
C26	0.17650 (5)	0.28672 (14)	0.40757 (11)	0.0413 (4)	
H26	0.202195	0.310881	0.400510	0.050*	
C27	0.16712 (5)	0.34448 (14)	0.49082 (11)	0.0415 (4)	
C28	0.17669 (5)	0.46539 (15)	0.50394 (12)	0.0450 (4)	
C29	0.19530 (6)	0.53161 (16)	0.44563 (14)	0.0567 (5)	
H29	0.201503	0.496668	0.394821	0.068*	
C30	0.20438 (7)	0.64609 (18)	0.46233 (17)	0.0721 (6)	
H30	0.216839	0.687805	0.423115	0.087*	
C31	0.19517 (8)	0.7009 (2)	0.53741 (19)	0.0803 (7)	
H31	0.201638	0.778734	0.548245	0.096*	
C32	0.17682 (7)	0.64135 (19)	0.59490 (17)	0.0725 (7)	
H32	0.170597	0.679086	0.644604	0.087*	
C33	0.16703 (5)	0.52252 (17)	0.58021 (13)	0.0540 (5)	
C34	0.14859 (6)	0.4597 (2)	0.64003 (14)	0.0643 (6)	
H34	0.142673	0.496707	0.690319	0.077*	
C35	0.13925 (6)	0.3460 (2)	0.62578 (14)	0.0633 (6)	
H35	0.126694	0.305819	0.665609	0.076*	
C36	0.14858 (5)	0.28885 (17)	0.55064 (12)	0.0522 (5)	
H36	0.141920	0.210833	0.541431	0.063*	
N1	0.17683 (4)	−0.00441 (12)	0.30412 (9)	0.0443 (4)	
N2	0.19688 (4)	0.10032 (13)	0.48422 (9)	0.0446 (4)	
O1	0.12667 (4)	0.14930 (13)	0.17294 (9)	0.0709 (4)	
O2	0.22409 (4)	0.15545 (12)	0.51946 (8)	0.0571 (4)	
O3	0.18748 (4)	0.00476 (11)	0.50940 (9)	0.0627 (4)	
O4	0.15134 (4)	0.33150 (10)	0.33224 (8)	0.0487 (3)	

### 6'-(nNphthalen-1-yl)-6a'-nitro-6',6a',6b',7',8',9',10',12a'-octahydro-2H-spiro[acenaphthylene-1,12'-chromeno[3,4-a]indolizin]-2-one (II) Atomic displacement parameters (Å^2^)

**Table:**

	*U*^11^	*U*^22^	*U*^33^	*U*^12^	*U*^13^	*U*^23^
C1	0.0576 (12)	0.0525 (11)	0.0461 (11)	−0.0046 (9)	0.0021 (9)	−0.0045 (9)
C2	0.0584 (13)	0.0660 (13)	0.0601 (13)	−0.0069 (10)	−0.0020 (10)	−0.0108 (11)
C3	0.0794 (17)	0.114 (2)	0.0852 (19)	−0.0180 (16)	−0.0215 (14)	−0.0094 (16)
C4	0.091 (2)	0.142 (3)	0.117 (3)	−0.032 (2)	−0.0279 (19)	−0.026 (2)
C5	0.0661 (17)	0.105 (2)	0.143 (3)	−0.0336 (16)	−0.0011 (19)	−0.044 (2)
C6	0.0606 (14)	0.0592 (14)	0.119 (2)	−0.0139 (11)	0.0230 (15)	−0.0269 (15)
C7	0.0746 (18)	0.0551 (15)	0.160 (3)	−0.0212 (13)	0.0402 (19)	−0.0172 (18)
C8	0.106 (2)	0.0475 (14)	0.138 (3)	−0.0099 (14)	0.037 (2)	0.0152 (15)
C9	0.0815 (16)	0.0490 (12)	0.0959 (18)	−0.0076 (11)	0.0127 (13)	0.0128 (12)
C10	0.0586 (12)	0.0402 (10)	0.0649 (13)	−0.0048 (9)	0.0127 (10)	−0.0030 (9)
C11	0.0490 (11)	0.0521 (12)	0.0761 (14)	−0.0082 (9)	0.0121 (10)	−0.0218 (11)
C12	0.0542 (11)	0.0382 (9)	0.0442 (10)	−0.0071 (8)	0.0031 (8)	−0.0002 (8)
C13	0.0747 (14)	0.0630 (13)	0.0510 (12)	−0.0012 (11)	0.0066 (10)	−0.0232 (10)
C14	0.0704 (14)	0.0788 (15)	0.0544 (12)	0.0128 (12)	0.0134 (11)	−0.0214 (11)
C15	0.0594 (12)	0.0819 (15)	0.0490 (11)	0.0065 (11)	0.0135 (9)	−0.0075 (11)
C16	0.0515 (11)	0.0555 (11)	0.0457 (10)	−0.0018 (9)	0.0096 (8)	−0.0066 (9)
C17	0.0497 (10)	0.0397 (9)	0.0320 (8)	−0.0028 (8)	0.0053 (7)	−0.0003 (7)
C18	0.0494 (10)	0.0364 (9)	0.0304 (8)	0.0001 (7)	0.0054 (7)	0.0016 (7)
C19	0.0505 (10)	0.0409 (9)	0.0399 (9)	−0.0043 (8)	0.0096 (8)	0.0022 (8)
C20	0.0476 (11)	0.0550 (11)	0.0435 (10)	0.0018 (9)	0.0062 (8)	−0.0027 (9)
C21	0.0538 (13)	0.0753 (15)	0.0722 (14)	−0.0007 (11)	0.0108 (11)	−0.0047 (12)
C22	0.0497 (13)	0.113 (2)	0.105 (2)	0.0131 (14)	0.0077 (13)	−0.0040 (18)
C23	0.0709 (18)	0.090 (2)	0.108 (2)	0.0287 (15)	0.0020 (15)	0.0058 (17)
C24	0.0750 (16)	0.0622 (13)	0.0709 (15)	0.0183 (12)	0.0017 (12)	0.0036 (11)
C25	0.0569 (12)	0.0527 (11)	0.0420 (10)	0.0078 (9)	0.0037 (9)	−0.0003 (9)
C26	0.0485 (10)	0.0374 (9)	0.0385 (9)	−0.0019 (8)	0.0075 (8)	0.0025 (7)
C27	0.0442 (10)	0.0399 (9)	0.0406 (9)	0.0026 (7)	0.0064 (8)	−0.0017 (8)
C28	0.0449 (10)	0.0399 (10)	0.0486 (10)	0.0050 (8)	0.0018 (8)	−0.0051 (8)
C29	0.0663 (13)	0.0409 (10)	0.0635 (13)	−0.0014 (9)	0.0117 (10)	−0.0022 (9)
C30	0.0810 (16)	0.0437 (12)	0.0903 (17)	−0.0069 (11)	0.0079 (13)	0.0020 (12)
C31	0.0918 (18)	0.0391 (12)	0.104 (2)	0.0025 (11)	−0.0069 (15)	−0.0148 (13)
C32	0.0802 (16)	0.0533 (13)	0.0797 (16)	0.0144 (12)	−0.0028 (13)	−0.0254 (12)
C33	0.0525 (11)	0.0490 (11)	0.0578 (12)	0.0125 (9)	−0.0005 (9)	−0.0120 (9)
C34	0.0679 (14)	0.0736 (15)	0.0543 (12)	0.0145 (11)	0.0183 (11)	−0.0170 (11)
C35	0.0673 (13)	0.0734 (15)	0.0537 (12)	0.0045 (11)	0.0240 (10)	−0.0015 (11)
C36	0.0613 (12)	0.0479 (11)	0.0494 (11)	0.0010 (9)	0.0142 (9)	−0.0020 (9)
N1	0.0514 (9)	0.0416 (8)	0.0400 (8)	−0.0027 (7)	0.0073 (7)	−0.0072 (6)
N2	0.0598 (10)	0.0397 (8)	0.0342 (8)	0.0023 (7)	0.0069 (7)	−0.0012 (7)
O1	0.0850 (10)	0.0700 (10)	0.0530 (8)	−0.0173 (8)	−0.0057 (7)	0.0154 (8)
O2	0.0614 (8)	0.0619 (9)	0.0445 (7)	−0.0041 (7)	−0.0042 (6)	−0.0052 (6)
O3	0.0911 (10)	0.0452 (8)	0.0497 (8)	−0.0023 (7)	0.0028 (7)	0.0117 (6)
O4	0.0599 (8)	0.0432 (7)	0.0428 (7)	0.0028 (6)	0.0073 (6)	0.0067 (5)

### 6'-(nNphthalen-1-yl)-6a'-nitro-6',6a',6b',7',8',9',10',12a'-octahydro-2H-spiro[acenaphthylene-1,12'-chromeno[3,4-a]indolizin]-2-one (II) Geometric parameters (Å, º)

**Table:**

C1—O1	1.205 (2)	C18—N2	1.526 (2)
C1—C2	1.474 (3)	C18—C26	1.538 (2)
C1—C12	1.583 (3)	C18—C19	1.542 (2)
C2—C3	1.371 (3)	C19—C20	1.499 (3)
C2—C11	1.396 (3)	C19—H19	0.9800
C3—C4	1.386 (4)	C20—C21	1.387 (3)
C3—H3	0.9300	C20—C25	1.389 (3)
C4—C5	1.361 (5)	C21—C22	1.382 (3)
C4—H4	0.9300	C21—H21	0.9300
C5—C6	1.416 (4)	C22—C23	1.374 (4)
C5—H5	0.9300	C22—H22	0.9300
C6—C11	1.398 (3)	C23—C24	1.358 (4)
C6—C7	1.402 (4)	C23—H23	0.9300
C7—C8	1.356 (4)	C24—C25	1.381 (3)
C7—H7	0.9300	C24—H24	0.9300
C8—C9	1.419 (3)	C25—O4	1.377 (2)
C8—H8	0.9300	C26—O4	1.449 (2)
C9—C10	1.354 (3)	C26—C27	1.516 (2)
C9—H9	0.9300	C26—H26	0.9800
C10—C11	1.404 (3)	C27—C36	1.364 (3)
C10—C12	1.514 (2)	C27—C28	1.433 (2)
C12—N1	1.454 (2)	C28—C29	1.410 (3)
C12—C19	1.572 (2)	C28—C33	1.424 (3)
C13—N1	1.468 (2)	C29—C30	1.366 (3)
C13—C14	1.507 (3)	C29—H29	0.9300
C13—H13A	0.9700	C30—C31	1.391 (4)
C13—H13B	0.9700	C30—H30	0.9300
C14—C15	1.504 (3)	C31—C32	1.356 (4)
C14—H14A	0.9700	C31—H31	0.9300
C14—H14B	0.9700	C32—C33	1.415 (3)
C15—C16	1.523 (3)	C32—H32	0.9300
C15—H15A	0.9700	C33—C34	1.403 (3)
C15—H15B	0.9700	C34—C35	1.353 (3)
C16—C17	1.514 (2)	C34—H34	0.9300
C16—H16A	0.9700	C35—C36	1.405 (3)
C16—H16B	0.9700	C35—H35	0.9300
C17—N1	1.461 (2)	C36—H36	0.9300
C17—C18	1.544 (2)	N2—O2	1.2174 (18)
C17—H17	0.9800	N2—O3	1.2233 (18)
			
O1—C1—C2	126.65 (19)	C26—C18—C19	114.61 (14)
O1—C1—C12	125.46 (17)	N2—C18—C17	105.74 (13)
C2—C1—C12	107.79 (17)	C26—C18—C17	111.64 (13)
C3—C2—C11	119.7 (2)	C19—C18—C17	104.29 (13)
C3—C2—C1	132.8 (2)	C20—C19—C18	113.32 (14)
C11—C2—C1	107.47 (17)	C20—C19—C12	113.13 (14)
C2—C3—C4	118.5 (3)	C18—C19—C12	104.94 (13)
C2—C3—H3	120.8	C20—C19—H19	108.4
C4—C3—H3	120.8	C18—C19—H19	108.4
C5—C4—C3	122.1 (3)	C12—C19—H19	108.4
C5—C4—H4	118.9	C21—C20—C25	118.24 (18)
C3—C4—H4	118.9	C21—C20—C19	121.96 (18)
C4—C5—C6	121.5 (3)	C25—C20—C19	119.73 (16)
C4—C5—H5	119.2	C22—C21—C20	120.4 (2)
C6—C5—H5	119.2	C22—C21—H21	119.8
C11—C6—C7	115.8 (2)	C20—C21—H21	119.8
C11—C6—C5	115.1 (3)	C23—C22—C21	119.8 (2)
C7—C6—C5	129.1 (3)	C23—C22—H22	120.1
C8—C7—C6	121.2 (2)	C21—C22—H22	120.1
C8—C7—H7	119.4	C24—C23—C22	121.0 (2)
C6—C7—H7	119.4	C24—C23—H23	119.5
C7—C8—C9	122.0 (3)	C22—C23—H23	119.5
C7—C8—H8	119.0	C23—C24—C25	119.3 (2)
C9—C8—H8	119.0	C23—C24—H24	120.3
C10—C9—C8	118.4 (3)	C25—C24—H24	120.3
C10—C9—H9	120.8	O4—C25—C24	118.78 (18)
C8—C9—H9	120.8	O4—C25—C20	119.99 (16)
C9—C10—C11	119.21 (19)	C24—C25—C20	121.2 (2)
C9—C10—C12	131.26 (19)	O4—C26—C27	109.11 (13)
C11—C10—C12	109.53 (17)	O4—C26—C18	106.57 (13)
C2—C11—C6	123.1 (2)	C27—C26—C18	119.13 (14)
C2—C11—C10	113.48 (17)	O4—C26—H26	107.2
C6—C11—C10	123.4 (2)	C27—C26—H26	107.2
N1—C12—C10	113.71 (15)	C18—C26—H26	107.2
N1—C12—C19	102.58 (13)	C36—C27—C28	119.11 (16)
C10—C12—C19	113.12 (15)	C36—C27—C26	123.32 (16)
N1—C12—C1	113.58 (15)	C28—C27—C26	117.49 (15)
C10—C12—C1	101.61 (15)	C29—C28—C33	117.74 (17)
C19—C12—C1	112.70 (14)	C29—C28—C27	123.77 (17)
N1—C13—C14	110.15 (16)	C33—C28—C27	118.49 (17)
N1—C13—H13A	109.6	C30—C29—C28	121.3 (2)
C14—C13—H13A	109.6	C30—C29—H29	119.4
N1—C13—H13B	109.6	C28—C29—H29	119.4
C14—C13—H13B	109.6	C29—C30—C31	120.6 (2)
H13A—C13—H13B	108.1	C29—C30—H30	119.7
C15—C14—C13	110.80 (18)	C31—C30—H30	119.7
C15—C14—H14A	109.5	C32—C31—C30	120.3 (2)
C13—C14—H14A	109.5	C32—C31—H31	119.8
C15—C14—H14B	109.5	C30—C31—H31	119.8
C13—C14—H14B	109.5	C31—C32—C33	120.8 (2)
H14A—C14—H14B	108.1	C31—C32—H32	119.6
C14—C15—C16	109.79 (17)	C33—C32—H32	119.6
C14—C15—H15A	109.7	C34—C33—C32	121.2 (2)
C16—C15—H15A	109.7	C34—C33—C28	119.54 (18)
C14—C15—H15B	109.7	C32—C33—C28	119.2 (2)
C16—C15—H15B	109.7	C35—C34—C33	121.09 (19)
H15A—C15—H15B	108.2	C35—C34—H34	119.5
C17—C16—C15	108.95 (15)	C33—C34—H34	119.5
C17—C16—H16A	109.9	C34—C35—C36	119.8 (2)
C15—C16—H16A	109.9	C34—C35—H35	120.1
C17—C16—H16B	109.9	C36—C35—H35	120.1
C15—C16—H16B	109.9	C27—C36—C35	121.92 (18)
H16A—C16—H16B	108.3	C27—C36—H36	119.0
N1—C17—C16	110.45 (14)	C35—C36—H36	119.0
N1—C17—C18	101.79 (13)	C12—N1—C17	106.72 (13)
C16—C17—C18	119.33 (14)	C12—N1—C13	116.30 (14)
N1—C17—H17	108.2	C17—N1—C13	114.36 (15)
C16—C17—H17	108.2	O2—N2—O3	124.22 (15)
C18—C17—H17	108.2	O2—N2—C18	116.50 (14)
N2—C18—C26	108.91 (13)	O3—N2—C18	119.18 (14)
N2—C18—C19	111.24 (13)	C25—O4—C26	112.30 (13)
			
O1—C1—C2—C3	−3.4 (4)	C12—C19—C20—C25	−99.28 (19)
C12—C1—C2—C3	179.9 (3)	C25—C20—C21—C22	1.4 (3)
O1—C1—C2—C11	174.7 (2)	C19—C20—C21—C22	−175.6 (2)
C12—C1—C2—C11	−2.0 (2)	C20—C21—C22—C23	−0.9 (4)
C11—C2—C3—C4	0.2 (4)	C21—C22—C23—C24	−0.5 (4)
C1—C2—C3—C4	178.2 (3)	C22—C23—C24—C25	1.2 (4)
C2—C3—C4—C5	−0.4 (5)	C23—C24—C25—O4	179.1 (2)
C3—C4—C5—C6	0.0 (6)	C23—C24—C25—C20	−0.7 (3)
C4—C5—C6—C11	0.7 (4)	C21—C20—C25—O4	179.57 (17)
C4—C5—C6—C7	−177.7 (3)	C19—C20—C25—O4	−3.4 (3)
C11—C6—C7—C8	−0.9 (4)	C21—C20—C25—C24	−0.6 (3)
C5—C6—C7—C8	177.4 (3)	C19—C20—C25—C24	176.42 (18)
C6—C7—C8—C9	1.6 (4)	N2—C18—C26—O4	−172.49 (12)
C7—C8—C9—C10	−1.3 (4)	C19—C18—C26—O4	−47.16 (18)
C8—C9—C10—C11	0.5 (3)	C17—C18—C26—O4	71.12 (17)
C8—C9—C10—C12	−179.6 (2)	N2—C18—C26—C27	−48.6 (2)
C3—C2—C11—C6	0.5 (3)	C19—C18—C26—C27	76.68 (19)
C1—C2—C11—C6	−177.97 (19)	C17—C18—C26—C27	−165.04 (14)
C3—C2—C11—C10	178.3 (2)	O4—C26—C27—C36	105.74 (18)
C1—C2—C11—C10	−0.2 (2)	C18—C26—C27—C36	−16.9 (2)
C7—C6—C11—C2	177.7 (2)	O4—C26—C27—C28	−70.94 (18)
C5—C6—C11—C2	−0.9 (3)	C18—C26—C27—C28	166.47 (15)
C7—C6—C11—C10	0.1 (3)	C36—C27—C28—C29	−179.01 (18)
C5—C6—C11—C10	−178.5 (2)	C26—C27—C28—C29	−2.2 (3)
C9—C10—C11—C2	−177.7 (2)	C36—C27—C28—C33	1.1 (2)
C12—C10—C11—C2	2.3 (2)	C26—C27—C28—C33	177.96 (15)
C9—C10—C11—C6	0.1 (3)	C33—C28—C29—C30	1.1 (3)
C12—C10—C11—C6	−179.87 (19)	C27—C28—C29—C30	−178.72 (19)
C9—C10—C12—N1	54.4 (3)	C28—C29—C30—C31	−0.5 (3)
C11—C10—C12—N1	−125.65 (17)	C29—C30—C31—C32	−0.4 (4)
C9—C10—C12—C19	−62.1 (3)	C30—C31—C32—C33	0.6 (4)
C11—C10—C12—C19	117.85 (17)	C31—C32—C33—C34	179.0 (2)
C9—C10—C12—C1	176.8 (2)	C31—C32—C33—C28	0.0 (3)
C11—C10—C12—C1	−3.2 (2)	C29—C28—C33—C34	−179.83 (18)
O1—C1—C12—N1	−51.1 (3)	C27—C28—C33—C34	0.0 (3)
C2—C1—C12—N1	125.63 (17)	C29—C28—C33—C32	−0.9 (3)
O1—C1—C12—C10	−173.6 (2)	C27—C28—C33—C32	178.98 (17)
C2—C1—C12—C10	3.1 (2)	C32—C33—C34—C35	180.0 (2)
O1—C1—C12—C19	65.0 (3)	C28—C33—C34—C35	−1.1 (3)
C2—C1—C12—C19	−118.25 (17)	C33—C34—C35—C36	0.9 (3)
N1—C13—C14—C15	−54.9 (2)	C28—C27—C36—C35	−1.3 (3)
C13—C14—C15—C16	58.2 (2)	C26—C27—C36—C35	−177.94 (17)
C14—C15—C16—C17	−58.6 (2)	C34—C35—C36—C27	0.3 (3)
C15—C16—C17—N1	56.9 (2)	C10—C12—N1—C17	−162.50 (15)
C15—C16—C17—C18	174.30 (16)	C19—C12—N1—C17	−40.00 (16)
N1—C17—C18—N2	85.76 (14)	C1—C12—N1—C17	81.93 (17)
C16—C17—C18—N2	−36.02 (19)	C10—C12—N1—C13	68.5 (2)
N1—C17—C18—C26	−155.94 (13)	C19—C12—N1—C13	−168.96 (15)
C16—C17—C18—C26	82.28 (19)	C1—C12—N1—C13	−47.0 (2)
N1—C17—C18—C19	−31.64 (15)	C16—C17—N1—C12	173.41 (14)
C16—C17—C18—C19	−153.42 (15)	C18—C17—N1—C12	45.67 (16)
N2—C18—C19—C20	130.92 (15)	C16—C17—N1—C13	−56.51 (19)
C26—C18—C19—C20	6.8 (2)	C18—C17—N1—C13	175.76 (15)
C17—C18—C19—C20	−115.54 (15)	C14—C13—N1—C12	−179.97 (17)
N2—C18—C19—C12	−105.17 (14)	C14—C13—N1—C17	54.9 (2)
C26—C18—C19—C12	130.73 (14)	C26—C18—N2—O2	−33.06 (19)
C17—C18—C19—C12	8.37 (16)	C19—C18—N2—O2	−160.32 (14)
N1—C12—C19—C20	142.03 (15)	C17—C18—N2—O2	87.05 (16)
C10—C12—C19—C20	−95.07 (18)	C26—C18—N2—O3	150.48 (15)
C1—C12—C19—C20	19.5 (2)	C19—C18—N2—O3	23.2 (2)
N1—C12—C19—C18	18.00 (16)	C17—C18—N2—O3	−89.41 (17)
C10—C12—C19—C18	140.90 (15)	C24—C25—O4—C26	137.77 (17)
C1—C12—C19—C18	−104.52 (16)	C20—C25—O4—C26	−42.4 (2)
C18—C19—C20—C21	−163.06 (17)	C27—C26—O4—C25	−63.81 (17)
C12—C19—C20—C21	77.6 (2)	C18—C26—O4—C25	66.03 (17)
C18—C19—C20—C25	20.0 (2)		

### 6'-(nNphthalen-1-yl)-6a'-nitro-6',6a',6b',7',8',9',10',12a'-octahydro-2H-spiro[acenaphthylene-1,12'-chromeno[3,4-a]indolizin]-2-one (II) Hydrogen-bond geometry (Å, º)

*Cg*1 is the centroid of the C6–C11 ring.

**Table:**

*D*—H···*A*	*D*—H	H···*A*	*D*···*A*	*D*—H···*A*
C13—H13*A*···O3^i^	0.97	2.59	3.413 (3)	143
C17—H17···O1	0.98	2.50	3.148 (2)	124
C32—H32···O1^ii^	0.93	2.59	3.318 (3)	135
C35—H35···*Cg*1^iii^	0.93	2.92	3.849 (2)	176

Symmetry codes: (i) *x*, −*y*, *z*−1/2; (ii) *x*, −*y*+1, *z*+1/2; (iii) *x*, −*y*, *z*+1/2.
